# Estimating Parameters of Speciation Models Based on Refined Summaries of the Joint Site-Frequency Spectrum

**DOI:** 10.1371/journal.pone.0018155

**Published:** 2011-05-26

**Authors:** Aurélien Tellier, Peter Pfaffelhuber, Bernhard Haubold, Lisha Naduvilezhath, Laura E. Rose, Thomas Städler, Wolfgang Stephan, Dirk Metzler

**Affiliations:** 1 Department of Biology II, Section of Evolutionary Biology, LMU University of Munich, Planegg-Martinsried, Germany; 2 Faculty of Mathematics and Physics, University of Freiburg, Freiburg, Germany; 3 Department of Evolutionary Genetics, Max-Planck-Institute for Evolutionary Biology, Plön, Germany; 4 Institute of Integrative Biology, Plant Ecological Genetics, ETH Zurich, Zurich, Switzerland; University of Cambridge, United Kingdom

## Abstract

Understanding the processes and conditions under which populations diverge to give rise to distinct species is a central question in evolutionary biology. Since recently diverged populations have high levels of shared polymorphisms, it is challenging to distinguish between recent divergence with no (or very low) inter-population gene flow and older splitting events with subsequent gene flow. Recently published methods to infer speciation parameters under the isolation-migration framework are based on summarizing polymorphism data at multiple loci in two species using the joint site-frequency spectrum (JSFS). We have developed two improvements of these methods based on a more extensive use of the JSFS classes of polymorphisms for species with high intra-locus recombination rates. First, using a likelihood based method, we demonstrate that taking into account low-frequency polymorphisms shared between species significantly improves the joint estimation of the divergence time and gene flow between species. Second, we introduce a local linear regression algorithm that considerably reduces the computational time and allows for the estimation of unequal rates of gene flow between species. We also investigate which summary statistics from the JSFS allow the greatest estimation accuracy for divergence time and migration rates for low (around 10) and high (around 100) numbers of loci. Focusing on cases with low numbers of loci and high intra-locus recombination rates we show that our methods for the estimation of divergence time and migration rates are more precise than existing approaches.

## Introduction

Understanding speciation processes is crucial in numerous fields including conservation biology, ecology, host-parasite co-evolution and human evolution [1]. According to the “biological species concept”, a species is defined as a group of interbreeding individuals that are reproductively isolated from other taxa [2]. Under this framework, the study of the speciation process focuses on the conditions leading to the emergence of reproductive isolation [3].

Allopatric population divergence is the classical scenario for isolation between populations [2]. In this model, two populations diverge in complete geographic isolation from one another. A second scenario considers divergence with continuing gene flow between populations, for example when species ranges abut (parapatry) or overlap following secondary contact, allowing for introgression. The latter model has been suggested to describe speciation events between human populations and ape species or sub-species [4], *Drosophila* species [5], and the wild tomato species *Solanum peruvianum* and *S. chilense* [6]. Key theoretical predictions have been generated to distinguish parapatric and allopatric population divergence based on genomic data [5], [7]. These show that under the model of parapatric separation greater variation in divergence time is expected across the genome compared to an allopatric model [5]. In other words, the variance of shared polymorphisms between populations can be used to distinguish between recent divergence without gene flow and an older split characterized by high levels of subsequent gene flow between populations [7]. However, to reliably use these variances for parameter estimation, data sets with large numbers of sequences are needed, which is a practical constraint in studies of many non-model organisms [8].

The most widely used general model of population divergence is the “isolation-migration” model [5]. This model has six parameters, assuming two populations are used: the splitting time, the effective population size of each extant population and of the ancestral population, and the rates of gene flow. Bayesian Markov-Chain Monte-Carlo (MCMC) methods to sample from the posterior distribution of the parameters given the full sequence data are implemented in the program IM and its successors IMa and IMa2 [5], [9], [10], [11]. Since the development and application of these methods to different species, a surprising number of cases indicate that speciation can occur in the presence of continual gene flow between incipient species [12]. However, existing implementations of these methods are limited to certain types of input data. For example, IM, IMa and IMa2 require that haplotypes are known and that there is no intra-locus recombination. This second assumption is particularly problematic in species in which the ratio of recombination to mutation rates is high, including *Drosophila melanogaster* [13] and wild tomato species [14], [15], [16]. In these species, recombination cannot be ignored since sequenced genomic fragments have experienced one or more recombination events [17]. In practice, researchers have excluded segments or haplotypes with evidence of recombination for inference of parameters using this method. This ostensible “solution” has two disadvantages. First, it introduces bias into parameter estimation because genealogies of samples without recombination tend to be shorter [5], [18]. Specifically, divergence time and current population sizes are shown to be overestimated, and ancestral population size is underestimated [18]. Second, for studies with few sequenced loci, the amount of data available for inference is significantly reduced, contributing to higher variances in parameter estimates.

**Figure 1:**
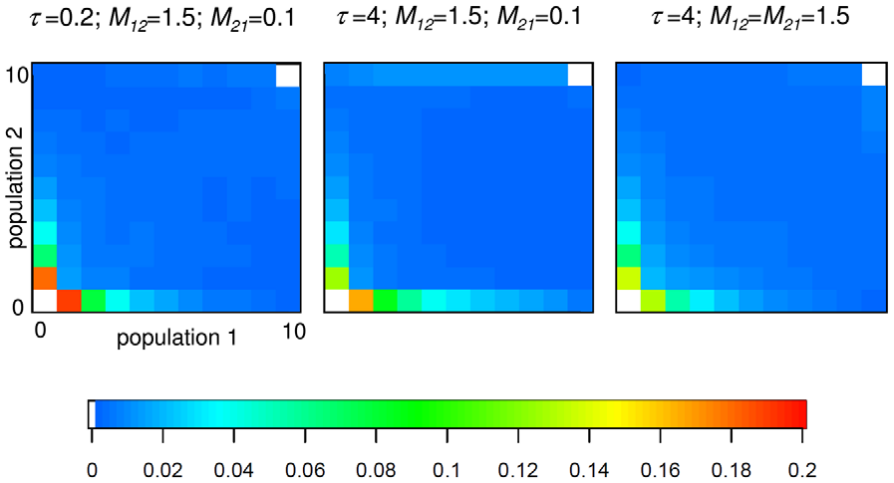
Three examples of joint site-frequency spectra for an Isolation-Migration model. An ancestral population of size *θ_A_* = 5 splits into two incipient populations (*θ_1_* = *θ_2_* = 5) at time *τ* = 0.2 or 4 in the past. 10 individuals are sampled from the two current populations and sequenced at 1,000 independent loci of 1,000 bp each. Intra-locus recombination occurs at a rate *ρ* = 0.02. The color legend indicates the proportion of polymorphisms in a given JSFS class. Migration rate from population 1 to 2 (*M_12_*), from population 2 to 1 (*M_21_*) and split time (*τ*) are indicated for each panel.

Other methods rely on summary statistics such as the joint site-frequency spectrum (JSFS) [19], which is an array *S* of dimension (*n_1_*+1)×(*n_2_*+1)−2 where entry *S_i,j_* is the number of polymorphic sites for which the derived state is found *i* times in the sample from population 1 and *j* times in the sample from population 2. For example, *S_2,3_* = 10 if 10 polymorphisms are found as doubletons in population 1 and as tripletons in population 2. For parameter estimation, Wakeley and Hey [19] summarized the JSFS by a vector *W* = (*W_1_*,*W_2_*,*W_3_*,*W_4_*) containing the number of private polymorphisms in species 1 and 2, respectively (*W_1_*, *W_2_*), fixed differences between species (*W_3_*), and shared ancestral polymorphisms (*W_4_*). Examples of JSFS expectation values are shown in Fig. 1 for various combinations of parameter values. Methods using summaries are aimed to be computationally faster than maximum-likelihood and Bayesian full-data methods while being reasonably accurate, especially when many independent loci are used [20]. The method MIMAR (MCMC estimation of the isolation-migration model allowing for recombination [4]) uses a variant of the Wakeley-Hey summary statistics *W*. Approximate Bayesian Computation (ABC) methods were also developed to estimate parameters of the isolation-migration model from summary statistics such as the amount of private polymorphisms and diversity per population and in the pooled sample (popABC [21]). A great advantage of ABC methods is that they can be implemented in a few days or weeks whereas the implementation of full-likelihood methods or Bayesian full-data MCMC algorithms may take months or years, though to check the quality of the summary statistics in the ABC might require additional time consuming simulations. More recently, Gutenkunst *et al.* [22] developed the method ∂a∂i, which takes into account the entire JSFS. Note that in ∂a∂i, all sites are considered to be independent, and the JSFS is calculated for all sites and not per locus contrary to other methods [22]. In this composite likelihood approach, the expectation values of the full JSFS are numerically computed using diffusion approximations.

The present study was motivated by research on non-model organisms, including, for example, two recently diverged species of wild tomatoes (*S. peruvianum* and *S. chilense*). Not only do these species appear to have recently diverged but gene flow may be on-going [6], [23]. The programs IM, IMa and IMa2 cannot be used due to high levels of intra-locus recombination. Furthermore, given the low number of genes sampled (7 to 13 in this case) methods based on the data summary *W* have limited power to distinguish between divergence in isolation and divergence with continuing gene flow. Since we wished to determine whether these two species split recently with no or negligible levels of gene flow, or split less recently, but diverged in the presence of gene flow, we realized that previously described methods were not adequate.

Our first aim is to show as a proof of concept that refining the summary of the JSFS to more classes results in improved estimates of divergence time and gene flow. For this purpose we decompose the class *W_4_* (shared polymorphisms) of the JSFS into further classes for singletons and doubletons shared between species (see Fig. 1). The rationale behind this new decomposition is that if gene flow between species has been low, as expected if the two species are distinct, there should be (i) few incidences of shared polymorphisms compared to the number of private polymorphisms per species [19], and (ii) recent migrants lead to an excess of low-frequency shared polymorphisms (singletons and doubletons) whose frequency over time is affected by drift. We observe in Figure 1 that indeed private polymorphism is in large excess compared to shared polymorphisms. However, under the assumption of constant gene flow [5], small variations in the low frequencies of shared polymorphism are indicative of the strength and symmetry of gene flow (Fig. 1). In the case of symmetric migration rates (gene flow from species 1 to species 2 equals that from species 2 to species 1) there is a symmetrical amount of shared low frequency polymorphism (singletons, doubletons) in both species (Fig. 1, third panel). On the other hand, if migration from species 1 to species 2 is high (and the opposite migration rate is low, Fig. 1 first and second panel) there is a higher proportion of shared polymorphism at low frequency in species 2, and a deficit of shared polymorphism at low frequency in species 1 (Fig. 1). We use the information from these differences in the amount of shared low frequency polymorphism in either species to estimate divergence time and gene flow using a simple likelihood ratio calculation method based on Hey and Nielsen [5]. We show that methods with more complex decompositions of *W* perform better than MIMAR.

The second aim is to develop a computationally efficient method designed for species with high levels of recombination (on the order of the mutation rate), which decreases the correlation across polymorphic sites. We neglect these dependencies and employ a composite likelihood approach based on a Poisson point process approximation of the JSFS, which significantly reduces the run time of the simulations. The parameter estimations are realized by local log-linear regression analysis. We demonstrate that this leads to a quantitative improvement of the use of the Wakeley-Hey summary statistics, because it allows the estimation of unequal directional gene flow between populations. Furthermore, computation time is much reduced compared to other methods. We show that our method is faster and gives more accurate estimates of divergence times and rates of gene flow than MIMAR, popABC, and ∂a∂i. However, for very recent divergence times (<0.1 *N_e_* generations) all methods overestimate divergence time and gene flow, although our more complex summary of the JSFS seems to be more robust than other methods. Importantly, we show that our composite likelihood methods based on the assumption of genealogically independent SNPs are also more accurate than previous methods when estimating parameters at low recombination rates. As a practical conclusion for the use of JSFS statistics, we apply our composite likelihood method to determine which JSFS decompositions yield the highest accuracy for estimating divergence and gene flow parameters. We provide this comparison for the case where 7 loci (approximately 300 to 400 SNPs as found in studies in wild tomato species [14], [23], [24]) or 100 sequenced loci (as available for some model organisms such as Drosophilids or primates [8]) are available.

## Methods

### 1. General model

We consider a neutral IM model in which an ancestral population splits into two populations that may exchange migrants. It is assumed that *n_1_* and *n_2_* alleles are sampled in the two populations and sequenced for a number of independently evolving loci (all loci have the same *n_1_* and *n_2_*). Following Wakeley and Hey [19], *μ* is the average mutation rate across loci and can be used to estimate the effective population sizes of the three populations (*N_A_*, *N_1_*, *N_2_*) if the scaled mutation rates *θ_A_* = 4*N_A_μ*, *θ_1_ = *4*N_1_μ* and *θ_2_ = *4*N_2_μ* can be estimated from the data. Note that as in Wakeley and Hey [19], *τ* is the estimated time of species divergence (in units of 2*N_1_* generations). The two migration rates *m_12_* and *m_21_* are defined as follows: *m_12_* is the fraction of population 2 that is replaced by migrants from population 1 each generation, and *vice versa* for *m_21_*. The migration parameter is rescaled as twice the number of individuals in a population replaced by migrants (backward in time) with *M_21_* = 4*N_1_m_21_* and *M_12_* = 4*N_2_m_12_*. In the current version, this model assumes that each locus is located on an autosome and follows the infinite-site mutation model with reciprocal recombination [25]. The coalescent simulations use Hudson's ms program [26]. Similar to Becquet and Przeworski [4], our model allows for intralocus recombination but not for gene conversion. The population recombination rate per base pair per generation is *c*. This value is assumed to be constant and known within a given locus and across all loci, *i.e.* we do not allow for variable recombination rates in the genome.

Following the description of the IM model by Hey and Nielsen [5], the posterior distribution of the parameters Θ = (*θ_A_*, *θ_1_*, *θ_2_*, *τ*, *M_12_*, *M_21_*, *c*) is
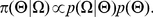
(1)where Ω is the data, *p*(Ω | Θ) is the likelihood of the vector of parameter values, Θ, and *p*(Θ) is its prior probability.

The full JSFS can be used to compare nucleotide sequence data of derived alleles from *n_1_* sequences from population 1 to *n_2_* sequences from population 2 [19]. It is assumed that an outgroup sequence is available and can be used to determine which allele is derived. Each derived allele is assigned to one cell of the JSFS depending on its frequency in the population. Note that *i* and *j* take integer values between 0 and *n_1_* and 0 and *n_2_*, respectively. Wakeley and Hey [19] and Hey and Nielsen [5] used summary statistics for parameter inference in the isolation-migration model. Formally, they are written as
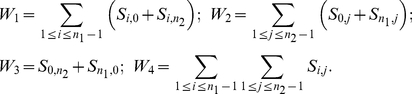
(2)Note that in MIMAR, Becquet and Przeworski [4] make use of an outgroup sequence to derive a slightly different set of four summary statistics for the frequencies of a derived allele:
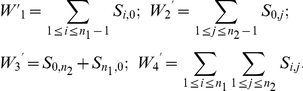



We demonstrate that using additional classes of the JSFS allows us to utilize more information than these original approaches, and improves the estimation of Θ. We present two methods that differ a) in the summary statistics used, *i.e.* different classes of the JSFS are used as summary statistics, and b) in the estimation procedure used to calculate the parameter values. To investigate the benefit of various sets of summary statistics for the joint estimation of divergence time and gene flow, we focus on estimating Θ = (*τ*, *M_12_*, *M_21_*) assuming that *θ_A_*, *θ_1_*, *θ_2_*, and *c* are known.

### 2. Maximum likelihood method

Our first approach is based on the maximum likelihood inference of the set of parameters Θ = (*τ*, *M_12_*, *M_21_*) [4], [7]. The data summaries are defined as a vector of four summary statistics extracted from the JSFS: *D*, *D′*, *D″*, D^*^. Our simplest summary of the JSFS, *D*, is a vector of 7 values (*D_k_*, *k* = 1,…,7) expanding the four classes *W_k_* (*k = *1,..,4) in Eq. 2. Additional classes relative to the Wakeley-Hey set are created by splitting each class of private polymorphisms to each species (*W_1_* and *W_2_*) and the fixed differences class (*W_3_*), by distinguishing whether the derived allele is fixed or absent in the other species. This results in the following relation between Eq. 2 and elements of *D*: *W_1_* = *D_1_+D_6_*, *W_2_* = *D_2_+D_7_*, *W_3_* = *D_3_+D_4_* and *W_4_* = *D_5_* (Appendix S1). The other vectors of summary statistics (*D′*, *D″*, D^*^) have more elements, 12 for *D′* and *D″* and 23 for D^*^, because singletons and doubletons in each population are included as new classes of shared polymorphism (see Appendix S1 for details). Compared to Nielsen and Wakeley [7] and Becquet and Przeworski [4], the class of shared polymorphisms between populations *W_4_* (Eq. 2) is further divided. The amount of information taken into account from the JSFS increases from *D* to D^*^, as shared low frequency and private polymorphisms are counted as separate elements of the summary statistics vector.

Following Eq. 1, the likelihood *L_D_*(Θ) = *p*(*D* | Θ) of the parameter combination Θ, for the given data summaries *D* (or similarly for *D′*, *D″*, D^*^) is an integral over all genealogies *G* (or Ancestral Recombination Graphs, ARG) [9], [27] as
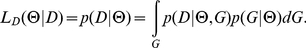
(3)The branch lengths of *G* are scaled in units of 2*N_1_* generations. Since the probability of the sequence data depends only on *G* and the mutation rate, we get:
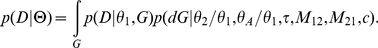
Thus, the likelihood *p*(*D* | Θ) can be approximated for each locus by generating a set of *I* genealogies *G_m_*, 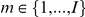, using Hudson's ms [26] as
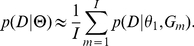
(4)In Eq. 4, *p*(*D* | *θ_1_*,*G_m_*) can be computed explicitly. The number *S_i,j_* of polymorphic sites of frequency *i* in population 1 and *j* in population 2 is Poisson distributed with mean *L_i,j_θ_1_*/2, where *L_i,j_* is the total length of ARG branches leading to *i* sequences in the first and *j* sequences in the second sample. Conditional on the genealogies, the probabilities of observing each element *D_k_* of the vector *D* are independent. The likelihood of the data for a given locus is approximated by
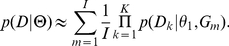
(5)Note that for the vector *D*, *K* = 7, but for *D′* and *D″*, *K* = 12, and for D^*^, *K* = 23.

A modified version of Hudson's ms is used to calculate the likelihood values for each simulated genealogy, and 10,000 genealogies were randomly drawn for each parameter combination. In the following, the maximum-likelihood methods based on these summaries are called D_1_ (using vector *D*), D_2_ (using vector *D′*), D_3_ (using vector *D″*) and D_4_ (using vector D*).

Since this method is not yet optimized for speed, the distribution of likelihood values is simply computed for values of Θ, *i.e. τ*, *M_12_* and *M_21_*, within a defined range. The maximum likelihood parameter values are obtained by local regression analysis using the locfit function available in the statistical software R (locfit package; [28]).

### 3. Composite likelihood method

Our second method is a variant of the method Jaatha, which is implemented as R code available from http://evol.bio.lmu.de/_statgen/software/jaatha. This method is computationally efficient because it takes advantage of the high recombination rate observed in *Drosophila* [13] and in some outcrossing plant species, including wild tomatoes [16]. This allows us to simplify the computation by treating the sites within and between loci as if they were independent. A further advance of this method is the improvement in estimation of rates of gene flow between populations, for example when migration rates are unequal.

Briefly, the method comprises three steps. First, summary statistics, *i.e.* classes of the JSFS, are calculated by coalescent simulations over the range of the three parameters to be estimated. Second, the three-dimensional parameter space is subdivided into 8×8×8 blocks. In each block, a log-linear regression (generalized linear model of Poisson type [29]) is fitted to the simulated data to describe for each of the JSFS classes how the expected number of mutations in this class depends on the *N_p_* parameters. Third, the composite likelihood of each block, given the observed values of JSFS summaries, is approximated using the fitted local log-linear regressions, and parameter estimates are obtained within the region with the highest likelihood. Note that the composite likelihood method is equivalent to the fitting of a multivariate Poisson distribution [30] to the summary statistics as a function of the genetic model parameters.

The parameters, *τ*, *M_12_*, and *M_21_*, of the isolation-migration model are estimated. Using Hudson's ms as coalescent simulator, we calculate summary statistics from the JSFS for numerous points on a grid in the parameter space (in this case a three-dimensional space). In the initial version of Jaatha, the JSFS is split into 23 elements constituting the vector *Ď_k_*, 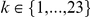. The vector *Ď* is similar to D* mentioned above as it considers classes of shared polymorphisms that are singletons or doubletons in both populations (*Ď_6_* in Appendix S1). However, *Ď* differs from D* through the addition of classes of shared polymorphism with nearly fixed frequencies (such as *n_1_* – 1, *n_1_* – 2, *n_2_* – 1, *n_2_* – 2). We give a detailed description of *Ď* in Appendix S1. In practice, simulations considered 40 different values for each parameter, and for each of the 40×40×40 = 64,000 parameter combinations, 10 coalescent simulations were performed and the vector *Ď* of summary statistics was stored.

Next, the three-dimensional space of parameters was divided into sub-regions of size *N_R_* for all three parameters. Each region contained *N_R_* ^3^ points characterized by the set of summary statistics *J*. In practice, we chose *N_R_* = 5, *i.e.* we subdivided the parameter space into 8×8×8 blocks each of which contained 5×5×5 different parameter combinations used in the simulation step. For each block and for each of the 23 summary statistics a log-linear Poisson regression model with the three parameters (*τ*, *M_12_*, and *M_21_*) as explanatory variables was fitted to the simulated data from 5×5×5×10 = 1,250 simulations (generalized linear model of Poisson type; [29]. For *x* = 1,…,5; *y* = 1,..,5 and *z* = 1,..,5 let *τ_x_*, *M_12,y_* and *M_21,z_* be the parameter values in a certain block. Then, *x* is an affine transformation of log(*τ_x_*) and the same holds for *y* with log(*M_12,y_*) and *z* with log(*M_21,z_*). Fitting the log-linear Poisson model for a certain block *b* and a certain summary *J_k_* requires the estimation of coefficients (*α_1,k_*, *α_2,k_*, *α_3,k_*, *α_4,k_*) such that the following equation holds for the expected value *d_k,x,y,z_* of *Ď_k_*

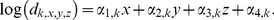
(6)or, equivalently,
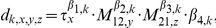
where parameter values of 0 are replaced by small positive values and *β_i,k_* is a transformation of *α_i,k_*. Given any parameter values *τ*, *M_12_*, and *M_21_* in the range of block *k*, the observed values of the summary statistic *Ď_k_* are assumed to be Poisson distributed with expected value 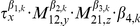. If *d_1,ϕ_* , *d_2,ϕ_* , …, *d_23,ϕ_* are the expected values of the 23 summary statistics for a certain combination *ϕ* = (*τ*, *M_12_*, *M_21_*) of parameter values and *F* = (*F_1_*, …, *F_23_*) are the observed values, then the Poisson model likelihood of *ϕ* is
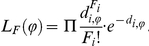
Note that Eq. 6 uses the logarithm of the parameter values to increase the resolution at low values, *i.e.* recent divergence time and low gene flow.

The first two steps are carried out independently of the observed data, and the most time-consuming part of the method is to fit regression models that describe how the expectation values of the summary statistics depend on the model parameters in the simulated data. The results of these steps can be reused to analyze data with similar sample sizes and parameter ranges. We have tried four different strategies for parameter estimation (called J_1_, J_2_, J_3_ and J_4_):

- J_1_. Only the 8×8×8 = 512 parameter combinations in the centers of the blocks are considered. Compute the Poisson model likelihood of each block center using the log-linear regression model. Output the block center with the highest value.
- J_2_. Output a weighted mean of the block centers. The weights are the Poisson model likelihoods as computed in J_1_.
- J_3_. For each block, start in the block center and numerically optimize the Poisson model likelihood within the block. Output the highest value that is found in any of the blocks.
- J_4_. Start an optimization in each block center. Allow the optimization search paths to change between the blocks. Near the block boundaries mixtures of the log-linear regression models fitted to the neighboring blocks are used to estimate the expected values of the summary statistics.

On a standard desktop computer, strategies J_1_ and J_2_ only take a few seconds, strategy J_3_ takes less than five minutes and strategy J_4_ takes 10 to 15 minutes for one data set. This requires that the log-linear model fitting has been performed in advance. Note that this step does not depend on the data. The fitting procedure takes about three to four days and the stored results can be re-used for data sets with the same sample sizes *n_1_* and *n_2_*.

### 4. Power analysis

#### i) Analysis for various JSFS coarsenings

We conducted a power analysis to compare the different coarsenings of the JSFS for estimating divergence time and detecting post-divergence gene flow. Sets of sampled loci were simulated under the IM model using Hudson's ms. We defined the simulated values of the model parameter as *τ_sim_*, *M_12-sim_*, and *M_21-sim_*. Then using the JSFS obtained for each set of simulation, we estimated the three parameters of the model (*τ_est_*, *M_12-est_*, and *M_21-est_*) using our maximum likelihood methods (D_1_–D_4_) and the composite method (J_1_–J_4_). For comparison, estimations were also computed using the MCMC-likelihood program MIMAR [4]. To make the methods comparable, MIMAR, D_1–4_ and J_1–4_ have identical fixed values for population sizes and recombination rate (*θ_A_*, *θ_1_*, *θ_2_*, and *c*) when estimating divergence and migration. The model underlying our simulation study is motivated by research on sequence variation in genes from non-model organisms for which few loci (here 7, each of length 1,000 bp) are available in two closely related species. However, our methods can also be applied to species for which numerous sequenced loci are available. In this case, the accuracy of the parameter estimates increases (see Fig. S11).

**Figure 2:**
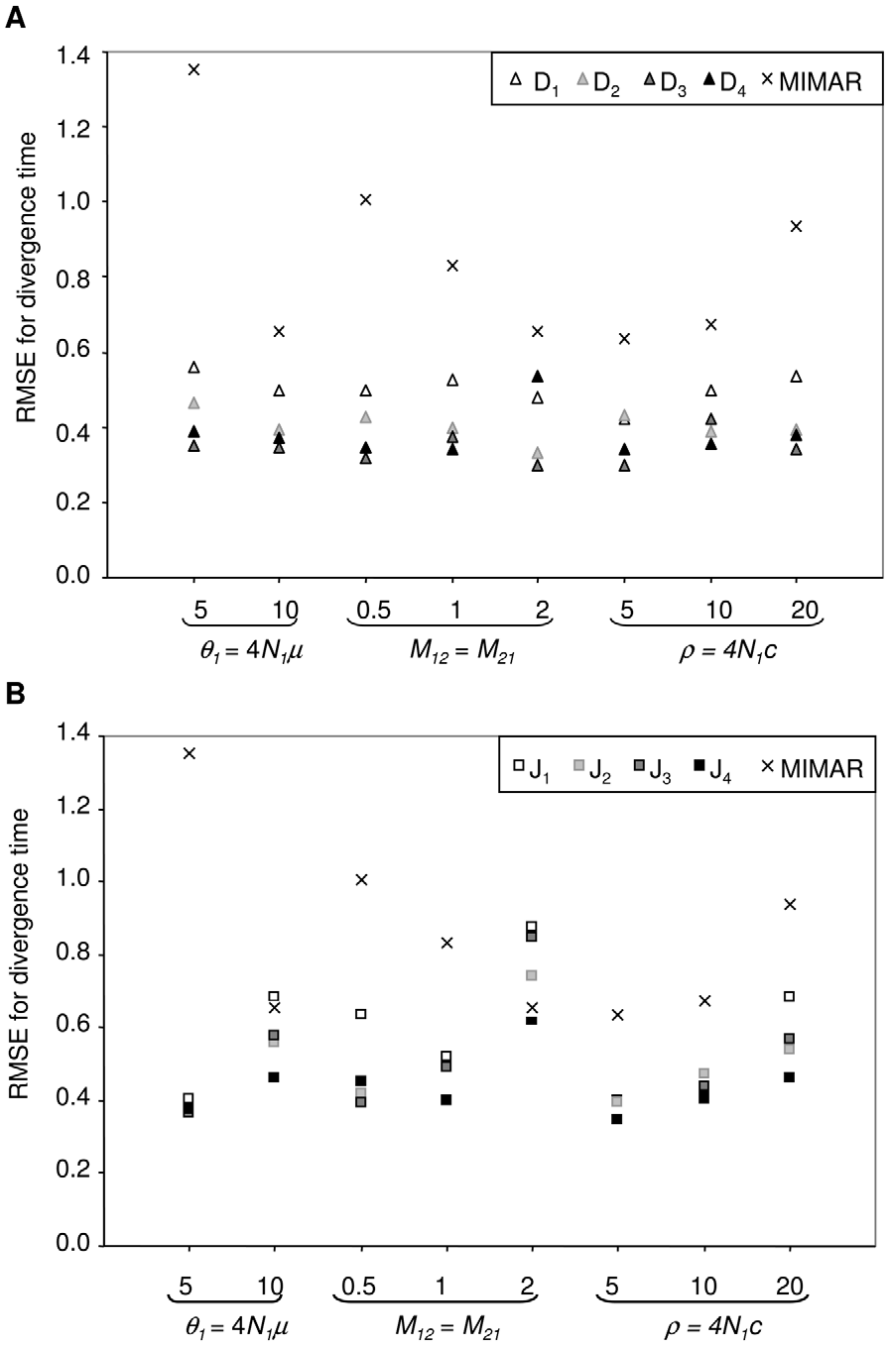
RMSE for the estimate of divergence time (*τ*) as a function of the population mutation rate (*θ*), values of simulated migration rate (*M_12_* = *M_21_*) and population recombination rate (*ρ*). The RMSE is computed across 140 datasets with divergence time fixed at *τ* = 0.1. (a) For the four maximum likelihood methods (D_1_–D_4_) and MIMAR, (b) for the four composite-likelihood methods (J_1_–J_4_) and MIMAR.

**Figure 3:**
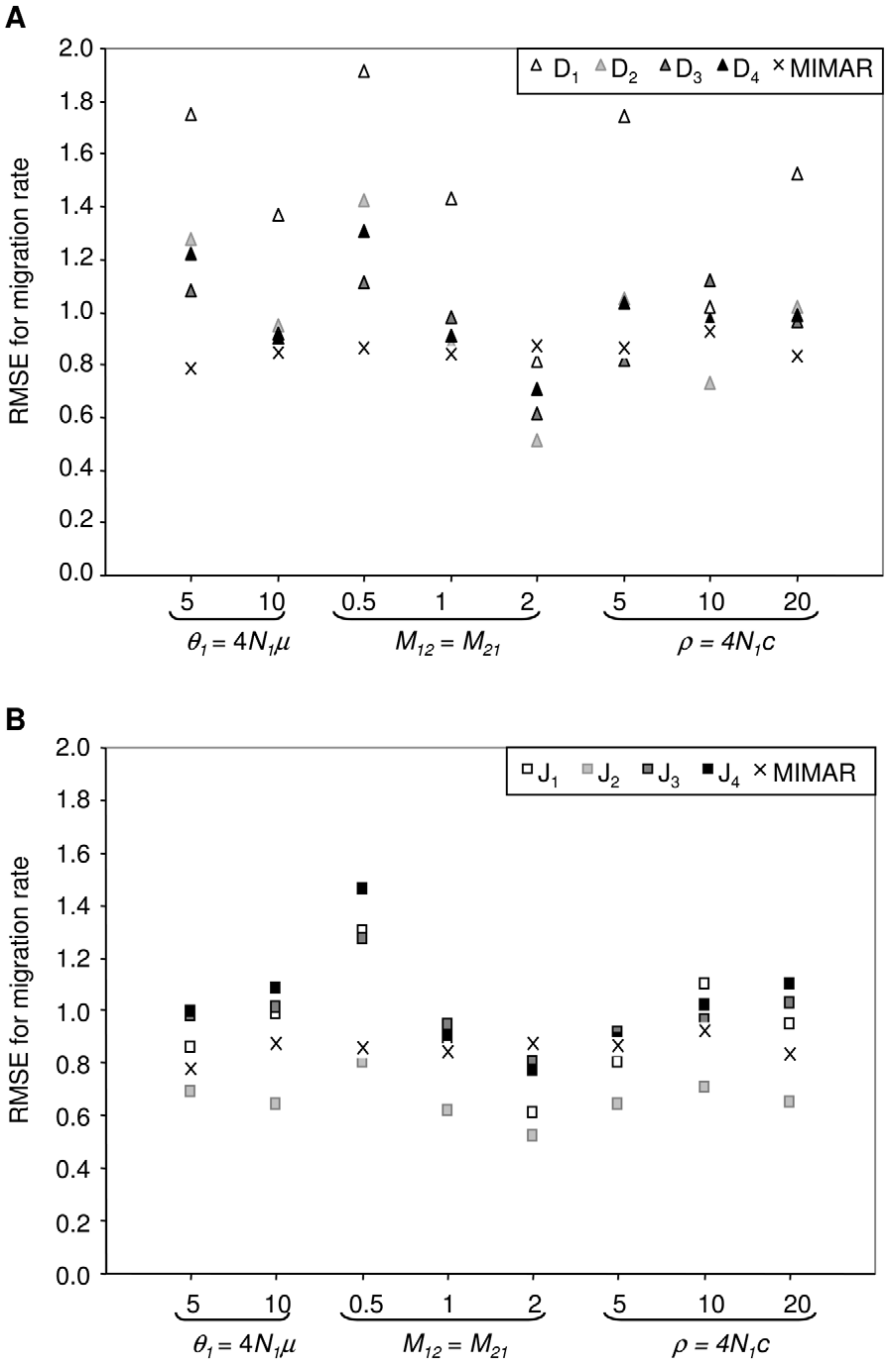
RMSE for the estimate of migration rate (*M_12_* = *M_21_*) as a function of the population mutation rate (*θ*), values of simulated migration rate (*M_12_* = *M_21_*) and population recombination rate (*ρ*). The RMSE is computed across 140 datasets with fixed divergence time at *τ* = 0.1. (a) for the four maximum likelihood methods (D_1_–D_4_) and MIMAR, (b) for the four composite-likelihood methods (J_1_–J_4_) and MIMAR.

We evaluated how the different coarsenings of the JSFS affect the accuracy of parameter estimates compared to MIMAR. For these analyses we fixed a recent divergence time to *τ = *0.1 but varied the migration rates (*M_12_*, *M_21_*) from very low (*M_12_ = M_21_* = 0.5) to intermediate (*M_12_* = *M_21_* = 2). Moreover, we investigated how other parameters of the model influence the accuracy of each method. Based on population sizes observed in wild tomatoes [14], [23], the mutation parameters *θ_A_*, *θ_1_*, *θ_2_* are assumed to be equal (*θ_A_* = *θ_1_ = θ_2_*), taking a value of 5 or 10. Similarly, the recombination rate *ρ* = 4*N_1_c* takes values of 5 (low *c*), 10 (intermediate *c*) or 20 (approximating high recombination). For each set of parameter values, 20 datasets of 7 loci were generated and analyzed using our maximum likelihood methods (D_1–4_), the composite method (J_1–4_) and MIMAR. MIMAR was run twice with two and 10 million steps of burn-in, the outputs being calculated based on 100,000 or 500,000 steps, respectively. Convergence to maximum likelihood values was assessed by a high rate of accepted steps, as recommended (over 10%; [4], [31]). The results of this analysis are shown in Figures 2 and 3 (and Figs. S1 and S2, Tables S1 and S2, Appendix S1).

#### ii) Analysis of robustness and speed

The second accuracy analysis deals with testing the robustness and speed of the composite method (J_1_–J_4_) by comparing performance with that obtained with MIMAR [4], the ABC implementation popABC [21], and the program ∂a∂i [22]. We generated 100 simulated data sets for a wide range of parameter values chosen at random. The divergence time was set from very recent (*τ* = 0.01) to ancient (*τ* = 9), migration rates were unequal (*M_12_*≠*M_21_*) each ranging from very low (*M* = 0.01) to high (*M* = 9). The mutation parameters *θ_A_* = *θ_1_* = *θ_2_* and the scaled recombination rate *ρ* = 4*N_1_c* were chosen at random between 5 and 20 per locus. The uniform priors for divergence time and migration rates are identical for our composite method (J_1_–J_4_), MIMAR, and popABC, and are defined as 0.01<*τ*<10 and 0.01<*M_12_*, *M_21_*<10. Note that all methods have identical fixed values for the population sizes and recombination rate (*θ_A_*, *θ_1_*, *θ_2_*, and *c*).

We used popABC to generate 300,000 simulations for each of the 100 data sets assuming fixed values of *ρ* and *θ_A_* = *θ_1_* = *θ_2_* for seven independent loci. The rejection and regression steps of the ABC were performed using the ABCreg code [32], with estimates of *τ*, *M_12_* and *M_21_* calculated as the mode of the best 3,000 (1%) simulations. Tests with popABC using all 22 possible summary statistics did not lead to reliable estimates. ABC methods can lack statistical power to estimate parameters when the number of summary statistics is too large [33], [34], because too few simulated datasets are close enough to the observed data, and the regression part of the ABC procedure cannot be realized. Therefore, we used fewer summary statistics. A first set of estimations are conducted based on six statistics from popABC closely related to the JSFS, *i.e.* for each species: the mean mutation frequency spectrum, an estimate of *F_ST_* based on segregating sites, and the number of private segregating sites [21]. A second set of estimations with 11 summary statistics was constructed by adding the number of segregating sites per species and for both species pooled, and the frequency of private polymorphisms. Finally, a third set of estimations with 14 statistics additionally comprised the number of different haplotypes in each species and for the pooled samples [21]. These 100 identical data sets were also analyzed using the ∂a∂i program [22]. However, we were unable to obtain reasonable parameter estimates from MIMAR. In fact, despite using 10 to 20 million burn-in steps, convergence to a maximum likelihood value for *τ*, *M_12_* and *M_21_* (fixing *ρ* and *θ_A_* = *θ_1_* = *θ_2_*) could not be obtained after more than 4 weeks of running. This is probably due to the wide range of priors for *τ*, *M_12_* and *M_21_* extending over several orders of magnitude (C. Becquet pers. comm.).

#### iii) Finding the best summary statistics

We looked for the best set of summary statistics, *i.e.* coarsenings *D*, *D′*, *D″*, D* or *Ď* of the JSFS, to be used for parameter estimation with our fast composite likelihood method. We ran methods J_1–4_ with these 5 different vectors of summary statistics and compared estimates with those obtained running methods J_1–4_ with the Wakeley-Hey vector of statistics (*W*). We analyzed the 100 simulated data sets of 7 loci (each of length 1,000 bp) with randomly chosen parameter values as described above. In addition, we performed a second analysis with simulated data sets of 100 independent loci of 1,000 bp each with parameters values in the same range as above (0.01<*τ*<9, *M_12_*≠*M_21_* and 0.01<*M*<9, *θ_A_* = *θ_1_* = *θ_2_* and *ρ* = 4*N_1_c* chosen at random between 5 and 20 per locus). The results of this analysis are shown in Figure 5 (and Figs. S6, S7, S8, S9, S10, Appendix S1).

**Figure 4:**
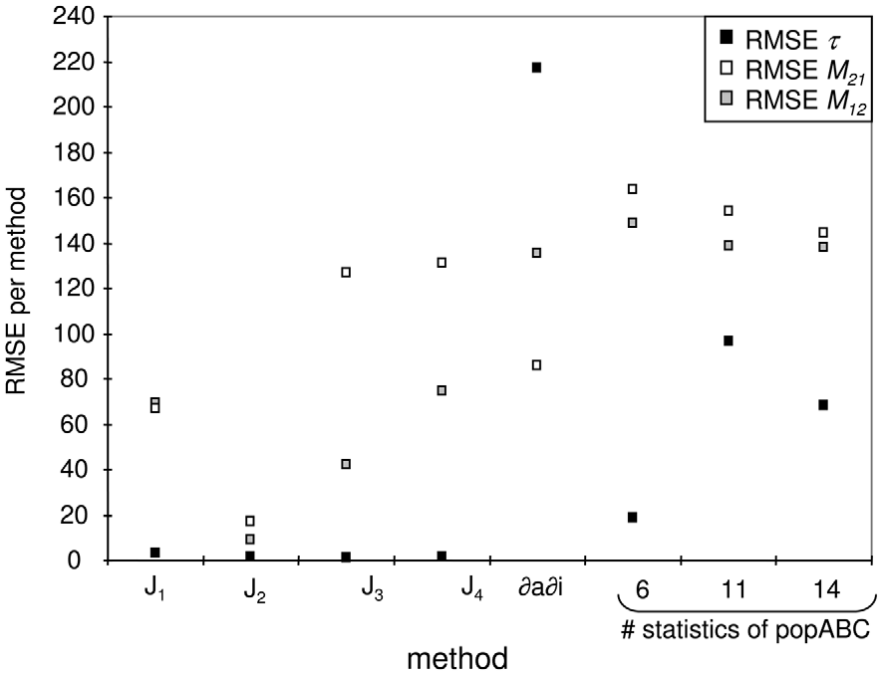
Comparison of RMSE for estimates of the divergence time and migration rates (*M_12_*≠*M_21_*) between methods. Results are shown for the four composite-likelihood methods (J_1_–J_4_), ∂a∂i, and for popABC with 6, 11 and 14 summary statistics (computed across 100 datasets).

**Figure 5:**
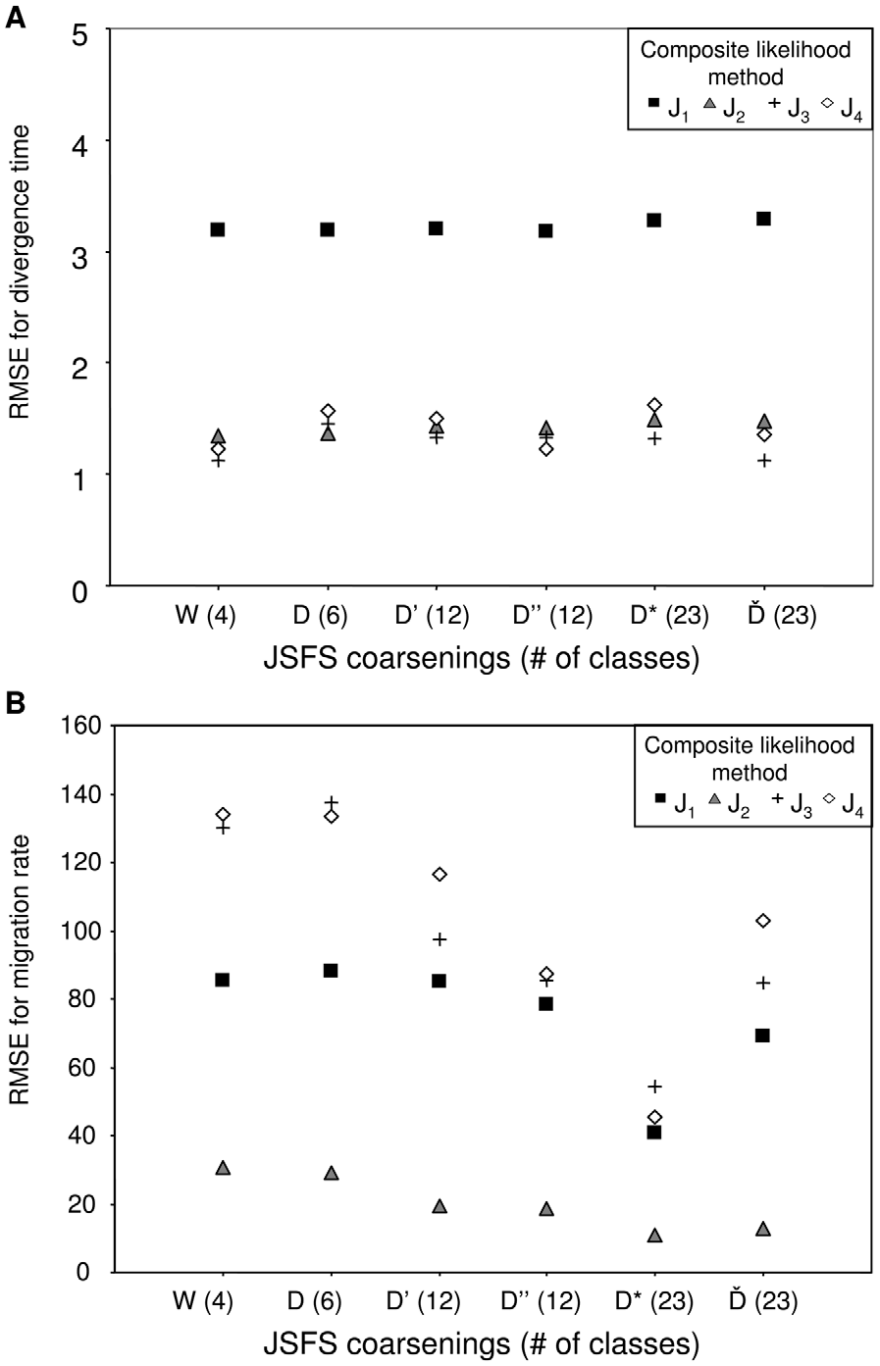
Power analysis of the various JSFS coarsenings to estimate divergence time and migration rates for 100 datasets of 7 loci. RMSE are computed for estimates of (a) the divergence time, and (b) migration rates (*M_12_*≠*M_21_*) for the four composite-likelihood methods (J_1_–J_4_) based on six vectors of summary statistics. The vector *W* is defined by the four Wakeley-Hey classes from Eq. 2, and other vectors *D*, *D′*, *D″*, D* and *Ď* are refined decompositions of the JSFS with higher numbers of classes.

#### iv) Statistical treatment

The results are presented in the format commonly used for power analyses. We report the mean of the estimate for each parameter value and three other statistics (see for example [35], [36]). The relative error (*RE*) is the relative difference between the estimated parameter value and the true parameter value that was used to simulate the data. For example, for the divergence time (*τ*), the relative error is *RE_τ_*:
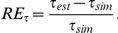
The root mean square error (RMSE) is the square root of the average squared difference (over *n_sim_* data sets) between the estimated value and the simulated value divided by the simulated value, and similarly, for *τ*:
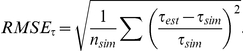
The Factor 2 (*F_2_*) is the proportion of data sets for which the estimated value (of *τ* or *M*) is at least half and at most twice the simulated value. Analyses of variance statistics were computed using the *glm* function, and multiple mean comparisons are based on Tukey's HSD test (confirmed by a Bonferroni test), as implemented in the R software ([28]; see Appendix S1, Tables S1 and S2 for details). We also analyzed the coverage of the methods, which is defined as the probability that the true parameter values are within the estimated 95% confidence range for *τ* and *M*. A possible approach to construct confidence ranges is based on the χ^2^-approximation for the distribution of log-likelihood ratios. In the case of two parameters, the confidence range consists of all parameter combinations for which the natural logarithm of the ratio of the maximum likelihood and the likelihood of the candidate values is smaller than 2.99 [37]. Coverage analyses were performed for this type of confidence range for the composite likelihood and the maximum likelihood methods, and for the credibility ranges reported by MIMAR based on 140 datasets of 7 loci (each 1,000 bp).

## Results

### 1. General results

All methods (maximum likelihood, composite likelihood, MIMAR, popABC, and ∂a∂i) showed variation in estimates of divergence time and, in particular, migration rates (Figs. 2, 3, 4 and Tables 1, 2). However, our methods showed the smallest relative error and RMSE for divergence time, resulting in good power to detect recent divergence (*τ* = 0.1; Figs. 2 and 3, Fig. S1). MIMAR significantly underestimated migration rates and overestimated divergence time compared to other methods (Figs. 2 and 3; Figs. S1 and S2).

**Table 1:** Relative error for estimates of divergence time with our composite likelihood methods, ∂a∂i, and popABC for 100 randomized datasets of 7 loci.

	Composite methods				∂a∂i	popABC		
	J_1_	J_2_	J_3_	J_4_		6 summary statistics	11 summary statistics	14 summary statistics
Minimum	−0.959	−0.953	−0.958	−0.959	−0.693	−0.875	−0.998	−0.998
Quartile 25%	−0.074	−0.157	−0.083	−0.094	0.107	0.569	−0.040	−0.770
Median	0.217	0.121	0.166	0.172	2.685	2.562	2.825	1.105
Quartile 75%	0.653	0.523	0.564	0.439	99.504	8.646	11.045	6.764
Maximum	30.404	11.894	7.001	8.59	957.562	139.88	775.128	578.51
Mean	0.747	0.434	0.454	0.498	96.953	8.146	23.170	15.635

**Table 2:** Relative error for estimates of the migration rate from population 1 to 2 (*M_12_*) with composite likelihood methods, ∂a∂i, and popABC for 100 randomized datasets of 7 loci.

	Composite methods				∂a∂i	popABC		
	J_1_	J_2_	J_3_	J_4_		6 summary statistics	11 summary statistics	14 summary statistics
Minimum	−0.996	−0.983	−0.998	−0.996	−0.968	−0.910	−0.989	−0.990
Quartile 25%	−0.509	−0.504	−0.56	−0.565	−0.072	−0.163	−0.797	−0.855
Median	−0.084	−0.07	−0.031	−0.101	0.371	9.175	−0.016	−0.201
Quartile 75%	0.801	0.69	0.464	0.499	3.883	57.874	20.738	17.902
Maximum	660.63	61.39	418.4	510.6	951.11	729.420	959.534	959.534
Mean	11.633	2.07	5.41	14.44	37.406	67.407	40.28	39.919

Over a large range of divergence times, from very recent (*τ* = 0.01) to very old (*τ* = 9), large overestimations were not common (relative error >10; Tables 1 and 2). However, migration rates were consistently overestimated by the composite likelihood methods, ∂a∂i, and popABC (*i.e.* relative error of 10 to 950; Table 1). Our methods J_1–4_ perform better than popABC and ∂a∂i in estimating both the divergence time and migration rates (Tables 1 and 2), and estimates of migration are always more accurate for high divergence times (*τ*>0.5) than for recent population splits (*τ*<0.5; Figs. S8 and S9).

An interesting, though expected, pattern is found when divergence time is fixed to a recent split, e.g. *τ* = 0.1. For our eight methods and MIMAR, a positive correlation is found between the relative error in estimates of divergence time and migration rates (Fig. S2). This means that when a given method over- or underestimates the divergence time, it also over- or underestimates the migration rate.

The estimates of divergence time and migration rates are only slightly affected by other population parameters, such as the mutation rate (*θ*) and the recombination rate (*ρ*). In fact, the relative error of the divergence time depends only on the method chosen and the population mutation rate. A significant interaction between method and *θ* is analyzed further by calculating the RMSE, in order to find which method performs better for a given value of *θ* (Fig. 2 and 3, Table S1). For all methods, the relative error of migration rates decreases when gene flow between populations increases (Fig. 3, Table S2).

### 2. Estimating divergence time

Our maximum likelihood methods D_3_ and D_4_ and composite-likelihood methods J_2_ and J_3_ perform better in estimating divergence time than other methods (MIMAR, D_1_, D_2_, J_1_, J_4_; see the lower RMSE in Fig. 2; Fig. S1). MIMAR shows increased accuracy in estimating *τ* as the migration rate (*M*) increases, reflecting the dependence between these parameter estimates (Fig. 3). This means that estimates of divergence time are improved by increasing the number of segregating sites, *i.e.* increasing *θ* (Fig. 2, Figs. S3 and S4). On the other hand, our methods do not show this trend (Figs. S6 and S7). On the contrary, the RMSE for divergence time increases as a function of *θ* for methods D_1–4_ (ANOVA in Table S1). According to the RMSE and Factor 2 values, our methods D_2_, D_3_, D_4_ and J_2_, J_4_ are the most accurate for estimating recent divergence time (Fig. 2, Figs. S3 and S4).

### 3. Estimating gene flow

Estimates of migration rates are generally less accurate than those of divergence time. The maximum likelihood methods D_1–4_ show greater variance in estimates than the composite methods J_1–4_ and MIMAR. However, MIMAR always underestimates the migration rate (Fig. 3). This consistent underestimation of migration rates by MIMAR results in small RMSE values because as the estimated migration rate goes to zero, the relative error, by definition, goes to −1 (Fig. S1b). Underestimation of migration rates by MIMAR is also revealed by the small Factor 2 values (Fig. S4). However, the lowest RMSE values are obtained for method J_2_ (Fig. 3b). All four composite methods show consistently low RMSE values at the three recombination rates tested (*ρ* in Fig. 3b). Maximum likelihood methods are more accurate for estimating migration rates when the true migration rate is large (lower RMSE and higher Factor 2, Fig. 3a). Overall, our eight methods estimate gene flow better when the rates are high.

### 4. Robustness and comparisons of methods

Our maximum likelihood methods are not sensitive to recombination, while MIMAR shows higher RMSE values in estimates of divergence time as recombination increases (Figs. 3a and 4a). Likewise, the RMSE increases for estimates of divergence time using our composite likelihood methods as *ρ* increases (although not significantly based on the ANOVA analysis; Table S1). Sensitivity to recombination is not found for estimates of migration rate (Fig. S7).

The ∂a∂i method tends to overestimate divergence time compared to other methods (Table 1). Relative error for estimates of very recent divergence times (*τ*<0.1) is high, although the median of the relative error rates is similar to results of popABC (Table 1). Compared to popABC, ∂a∂i is more accurate in estimating migration rates, demonstrating the statistical power gained by considering the maximum amount of information from the JSFS (Table 2, Fig. S5). However, the overall performance of ∂a∂i in estimating divergence time and migration rates is worse than that of our composite-likelihood methods (higher RMSE in Fig. 4, Fig. S5).

### 5. Advantage of using more than four JSFS based summary statistics and more loci

We demonstrate the benefit of using more than four statistics of the JSFS for estimating divergence time and migration rates. Methods relying on relatively few classes within the JSFS such as MIMAR and our maximum likelihood method D_1_ (with only 7 classes of the JSFS) tend to over- or underestimate divergence time and migration rates more often than the other maximum likelihood methods (D_2–4_; Figs. 1, 2 and 3). In fact, RMSE values for divergence time are higher for D_1_ and MIMAR compared to D_2–4_ (Fig. 2a), and higher for migration rate under D_1_ compared to D_2–4_ (Fig. 3a). Second, estimates from composite-likelihood methods show RMSE values that are several orders of magnitude lower for divergence time than those obtained with popABC, which relies on very limited information from the JSFS (Fig. 4). Running popABC with six statistics was the most accurate method to estimate divergence time, compared to using more statistics (11 and 14; Fig. 4). Third, JSFS-based summary statistics provide more accurate estimates (*i.e.* lower RMSE and higher Factor 2) of unequal migration rates between populations (*M_12_*≠*M_21_*) than do popABC statistics (Fig. 4, Tables 1 and 2).

Finally, our comparison of the different JSFS coarsenings using the composite likelihood method shows that estimates of migration rates are more accurate when considering the vectors *D″*, D* or *Ď* compared to vectors *W*, *D* and *D′* (Fig. 5b). The vectors *D″*, D* and *Ď* contain 12 or 23 summary statistics from the JSFS, whereas *W* and *D* have only four and six. Note, however, that the RMSE for estimating divergence time is not affected by the choice of summary statistics (Fig. 5a). For datasets with seven loci, the composite likelihood method J_2_ performs better for all coarsenings of the JSFS, as shown by the dramatic decrease of the RMSE for migration rates in Figure 5b. For datasets with 100 loci, estimates of divergence time and especially migration rates are improved compared to the seven loci case (RMSE values in Fig. S11 and Fig. 5). However, note that for 100 loci, the best estimates of migration rates are obtained with our composite likelihood methods J_3–4_ using coarsenings with 23 statistics (D* or *Ď*; Figure S11b).

## Discussion

There is growing interest in speciation models and the estimation of the parameters of these models from DNA sequence data. To perform such statistical inferences requires the use of efficient sets of summary statistics to apply to the increasing amount of sequence data [34]. Recent theoretical studies have focused on examining the biases in estimating parameters of the isolation-migration model [5], [9] when some key assumptions are violated, such as constant levels of post-divergence gene flow, the absence of population structure, and no migration from an unsampled species [4], [18]. Following the approach pioneered by the authors of the MIMAR software, we developed methods to tackle two limitations of existing estimation procedures: the pervasive problem of intra-locus recombination and the often limited number of loci sequenced (around 10) and individuals sampled. These two factors typically represent severe limitations for studying recent speciation in non-model species, such as wild tomatoes [6], [23].

The JSFS is a summary of polymorphism data that contains information about the parameters of the isolation-migration model [5], [7], [19]: the divergence time (*τ*), the population sizes of the two extant populations (*θ_1_* and *θ_2_*), the ancestral population size (*θ_A_*), and the migration rates between populations (*M_12_* and *M_21_*). The likelihood methods of Nielsen and Wakeley [7] and Becquet and Przeworski [4] use four classes of the JSFS to estimate parameters. In addition to these four classes, our coarsenings *D′*, *D″*, D* and *Ď* take low-frequency polymorphisms that are shared between populations into account. We show that this provides a significant improvement for estimating the divergence time and gene flow between populations under recent divergence and across a range of intra-locus recombination rates.

Reliable estimates of migration rate and divergence time are linked to variances in the four classes of the JSFS [4], [7]. Thus, data sets with many sequences are needed [8]. When only a few loci are sampled, estimates of divergence time and gene flow are correlated [5]. Our novel sets of JSFS-based summary statistics allow to improve the joint estimates of these two parameters, especially when only a small number of loci and SNPs are sampled. In other words, when the information content of the data is limited, one should avoid using a small part of the JSFS and a few summary statistics, because too much information is disregarded (see Fig. 1). Especially in the case of recent divergence, our methods are more accurate than previous ones to disentangle migration from divergence by considering more summary statistics for low-frequency shared polymorphisms. Indeed, if gene flow occurs between diverging species, the rate of gene flow should be low, and this would be reflected by a higher number of shared low-frequency polymorphisms. The use of a more complex summary of the JSFS thus enhances the accuracy of joint parameter estimates of the IM model for any number of sampled loci (for example 7 or 100). Note that in our examples, the simulated 7 loci contain approximately 350 SNPs to emulate date sets obtained from *Drosophila* and wild tomatoes [14], [23], [24]. This number of SNPs in combination with high recombination rates explains the improvement of statistical accuracy shown by our methods compared to previous ones, except for very recent divergence (where all methods fail).

Our results show in addition that the coverage of the maximum likelihood methods (varying from 64 to 86%) is higher than that of the composite likelihood methods (50%) and MIMAR (around 10%). These results indicate that the MIMAR runs may have converged on local optima and confirm that the chi-square approximation for confidence intervals is applicable to our composite likelihood method [37]. However, even for our maximum-likelihood method, coverage stays below the target value of 95%. We thus advocate that general approaches like parametric bootstrapping would have to be applied for hypothesis testing and to compute confidence intervals in our newly proposed estimation methods [38].

A second quantitative improvement is achieved by developing a simulation-based composite likelihood method that considerably reduces the time of computation compared to MIMAR and our maximum likelihood methods. These methods, as well as full likelihood procedures such as IM [5], require extensive search of the parameter space, which is very time-consuming. Typically, our maximum likelihood methods and MIMAR must run for three to four weeks for a single data set on a standard desktop computer. On a similar machine, popABC can be run for three to four days to generate a table of 300,000 simulations. The rejection and regression steps are then instantaneous. Our composite-likelihood methods require three to four days to generate the JSFS grid of parameter combinations. However, an advantage is that this grid can be used for multiple analyses with the same type of model and identical sample sizes. Note also that our priors can be used for any number of loci, so that the runtime of our composite-likelihood methods does not scale with the number of loci. ABC methods (*e.g.*, popABC) can also re-use a given simulated parameter space if the data sets to be analyzed have identical prior distributions.

Our methods J_2–4_ (with coarsenings D* or *Ď*) provide the most accurate estimates of migration rate. The assumption of independence of sites does not affect the power of these methods over a range of recombination rates (∂a∂i shows a similar behavior). This indicates that methods which take intra-locus recombination into account are also valid when rates of recombination are low [4]. However, the converse is not true. Methods based on the full likelihood analysis of haplotypic data which assume no intra-locus recombination [5], [9] are biased if recombination is present [4], [18], [31]. Another advantage of our composite-likelihood method is that unequal rates of gene flow between diverging species can be estimated (as does ∂a∂i, [22]). Unequal migration rates introduce an asymmetry in the JSFS between the expected numbers of shared low-frequency polymorphisms in each species [22]. Thus, unequal rates of gene flow between species can only be estimated by using a more complex summary of the JSFS than the four Wakeley-Hey summary statistics included in our *W* vector (*W_1_*, *W_2_*, *W_3_*, *W_4_*).

Estimates of divergence time and migration rates with the ABC method clearly suffer from large overestimates (relative error >50). For popABC extreme overestimates of the divergence time occur when the true value is very low (*τ*<0.1 in Tables 1 and 2, Fig. S10), independent of the migration rate. Similarly, *M_12_* (or *M_21_*) is biased under low migration (*M_12_* or *M_21_*<0.1), independent of the divergence time. In contrast, when using the composite likelihood methods (J_1–4_), large relative errors are observed for estimates of the migration rate *M_12_* if the true migration rate is low (*M_12_*<0.1) and the divergence time is very recent (*τ*<0.1, Figs. S8 and S9). This means that the summary statistics (whether all 22 or a subset) used in the ABC framework of popABC are not sufficiently sensitive to obtain precise joint estimates of gene flow and divergence time. Furthermore, note as well that popABC does not incorporate an outgroup, which might also explain the reduced information contained in the summary statistics.

We also notice that inaccurate estimation of parameters with popABC following the regression is due to wide posterior distributions. The mode of the posterior estimated by ABCreg [32] was always contained in the posterior calculated by the rejection algorithm in popABC (also based on the best 1% of the simulations; [21]). However, when posterior distributions have wide 95% credibility intervals, the mode computed after the regression step overestimates the true value, especially for migration rates. Wide posterior distributions for divergence time and migration rate estimates occurred when either of these parameters was small (recent divergence *τ*<1 or small migration *M*<0.1). Estimates obtained with 14 summary statistics are more accurate than those obtained with 11, although they differ only by the inclusion of haplotype diversity in each population and over pooled populations (Fig. 4). This highlights the fact that information contained in haplotype structure helps to disentangle the effects of migration and divergence on genetic diversity. We suggest that an ABC method using more classes of the JSFS such as our vectors D* or *Ď* (in addition to haplotype diversity), would show better inference of recent divergence times and gene flow, and might be robust over a range of recombination rates.

Finally, we find less accurate estimates of divergence time and gene flow with ∂a∂i than with our composite likelihood methods (J_1–4_; Fig. 4). This is surprising since ∂a∂i is also a composite likelihood approach, in which the expected values of the full JSFS are computed numerically via a diffusion approximation [22]. This method overestimates divergence time, especially for very recent divergence events (*τ*<0.1), but estimations of migration rate are in line with results from our composite methods and popABC (Table 1 and 2). In other words, when only a few loci are sampled and divergence is recent, the amount of information contained in the JSFS appears to limit the precision of the inferred gene flow parameters. We suggest that our composite-likelihood method based on local regression is more robust to the violation of the assumption that all SNPs are independent than are methods based on diffusion approximations. This would explain the lower accuracy of ∂a∂i compared to our methods. Details of the behavior of ∂a∂i when estimating parameters are, however, beyond the scope of this paper.

In conclusion, we have shown that existing statistical methods to infer speciation parameters in the isolation-migration framework based on the JSFS are improved by more extensive partitioning of the JSFS classes. We have developed a composite-likelihood method that allows to distinguish the signatures of young divergence from those of older divergence time but with recurrent gene flow between populations; these methods are particularly suitable for species with intra-locus recombination and a limited amount of data (less than 20 loci). When analyzing data from two or more diverging populations or species, it should be kept in mind that departures from the stringent model assumptions [5], [12], [19], such as drawing inference from coding sequences or introns with different selection regimes between species [24], may bias estimates of divergence time, gene flow, and population sizes [18], [31].

## Supporting Information

Appendix S1 **Supplementary information.**
(PDF)

Figure S1 **Relative error for estimates of (a) the divergence time (** ***τ*** **) and (b) the migration rate (** ***M*** **=** ***M_12_*** **=** ***M_21_*** **), for the maximum likelihood methods (D_1_–D_4_), MIMAR and the composite-likelihood methods (J_1_–J_4_).** Relative error is calculated as (*τ_est_−τ_sim_*)/*τ_sim_* where *τ_est_* is the estimated value and *τ_sim_* is the simulated value. Groups with significant differences between means following multiple comparisons (Tukey HSD test at 0.05) are indicated by letters for each method (group *a* for the smallest mean). Values that are more than 1.5 times the nearest interquartile range (25% or 75%) are displayed as diamonds, those more than 3 times are displayed as stars.
(TIF)

Figure S2 **Analysis of regression between errors in estimates of migration rate (** ***M_12_ = M_21_*** **) and divergence time** ***τ*** **for the 9 methods tested.** (a) D_1–4_ for the maximum likelihood methods, (b) J_1–4_ for the composite likelihood methods and (c) for MIMAR. Positive (negative) relative error indicates over (under)-estimation of the parameter. Regression coefficients and p-values are calculated using the *lm* function in the R software. P-values indicate the significance of the test whether the slope of the linear regression is zero.
(TIF)

Figure S3 **Factor 2 as a percentage of the estimates of divergence time (** ***τ*** **) in the range** ***τ_sim_*** **/2<** ***τ_est_*** **<** ***τ_sim_*** **×2 as a function of the population mutation rates (** ***θ*** **), values of simulated migration rates (** ***M_12_ = M_21_*** **) and population recombination rates (** ***ρ*** **).** The Factor 2 (*F_2_*) is the proportion of data sets for which the estimated value (of *τ* or *M*) is at least half and at most twice the simulated value: (a) for the four maximum likelihood methods (D_1_–D_4_) and MIMAR, (b) for the four composite-likelihood methods (J_1_–J_4_) and MIMAR.
(TIF)

Figure S4 **Factor 2 as a percentage of the estimates of migration rate (** ***M*** **=** ***M_12_ = M_21_*** **) in the range** ***M_sim_*** **/2<** ***M_est_*** **<** ***M_sim_*** **×2 as a function of the population mutation rate (** ***θ*** **), values of simulated migration rates (** ***M_12_ = M_21_*** **) and population recombination rates (** ***ρ*** **).** (a) For the four maximum likelihood methods (D_1_–D_4_) and MIMAR, (b) for the four composite-likelihood methods (J_1_–J_4_) and MIMAR.
(TIF)

Figure S5 **Factor 2 for estimates of the divergence time and migration rates (** ***M_12_*** **,** ***M_21_*** **) for the four composite-likelihood methods (J_1_–J_4_), ∂a∂i and for popABC with 6, 11 and 14 summary statistics (computed over 100 datasets).**
(TIF)

Figure S6 **Distribution of relative error for (a) divergence time and for (b) migration rate depending on the population mutation rate (** ***θ*** **) for composite-likelihood method J_4_.** For clarity, only relative errors lower than 15 are shown in (b).
(TIF)

Figure S7 **Distribution of the relative error of (a) divergence time and of (b) migration rate depending on the population recombination rate (** ***ρ*** **) for composite-likelihood method J_4_.** For clarity, only relative errors lower than 15 are shown in (b).
(TIF)

Figure S8 **Relative error for estimation of migration rate depending on the simulated value of the migration rate (** ***M_12_*** **in blue and** ***M_21_*** **in red) for composite method J_2_.** (a) For simulated divergence times less than 0.5, and (b) for simulated divergence times greater than 1. Note the difference in scale of the y-axes between (a) and (b).
(TIF)

Figure S9 **Relative error in the estimation of the migration rate (** ***M_12_*** **in blue and** ***M_21_*** **in red) depending on the simulated value of the migration rate for composite likelihood method J_4_.** (a) For simulated divergence times smaller than 0.5, and (b) for simulated divergence times greater than 1. Note the difference in scale of the y-axes between (a) and (b).
(TIF)

Figure S10 **Relative error in the estimation of migration rate depending on the simulated value of the migration rate (** ***M_12_*** **in blue and** ***M_21_*** **in red) for popABC estimates with 6 summary statistics.** (a) For simulated divergence times smaller than 0.5, and (b) for simulated divergence times greater than 1.
(TIF)

Figure S11 **Power analysis of the various JSFS coarsenings to estimate divergence time and migration rates for 100 datasets of 100 loci.** RMSE are computed for estimates of the (a) divergence time (*τ*) and (b) migration rates (*M_12_*≠*M_21_*) for the four composite-likelihood methods (J_1_–J4) based on six vectors of summary statistics with different numbers elements. The vector *W* is defined by the Wakeley-Hey 4 classes from Eq. 2, and other vectors *D*, *D′*, *D″*, D* and *Ď* are refined decompositions of the JSFS with higher number of classes.
(TIF)

Table S1 **ANOVA table of analysis of error in the estimation of divergence times (** ***τ*** **).**
(PDF)

Table S2 **ANOVA table of analysis of error in the estimation of migration rates (** ***M_12_ = M_21_*** **).**
(PDF)

## References

[1] Hey J (2006). On the failure of modern species concepts.. Trends Ecol Evol.

[2] Mayr E (1963). Animal species and evolution.

[3] Coyne JA, Orr HA (2004). Speciation.

[4] Becquet C, Przeworski M (2007). A new approach to estimate parameters of speciation models with application to apes.. Genome Res.

[5] Hey J, Nielsen R (2004). Multilocus methods for estimating population sizes, migration rates and divergence time, with applications to the divergence of *Drosophila pseudoobscura* and *D. persimilis*.. Genetics.

[6] Städler T, Roselius K, Stephan W (2005). Genealogical footprints of speciation processes in wild tomatoes: Demography and evidence for historical gene flow.. Evolution.

[7] Nielsen R, Wakeley J (2001). Distinguishing migration from isolation: A Markov chain Monte Carlo approach.. Genetics.

[8] Wang Y, Hey J (2010). Estimating divergence parameters with small samples from a large number of loci.. Genetics.

[9] Hey J, Nielsen R (2007). Integration within the Felsenstein equation for improved Markov chain Monte Carlo methods in population genetics.. Proc Natl Acad Sci USA.

[10] Won YJ, Hey J (2005). Divergence population genetics of chimpanzees.. Mol Biol Evol.

[11] Hey J (2010). Isolation with migration models for more than two populations.. Mol Biol Evol.

[12] Hey J (2006). Recent advances in assessing gene flow between diverging populations and species.. Curr Opin Genet Dev.

[13] Andolfatto P, Wall JD (2003). Linkage disequilibrium patterns across a recombination gradient in African *Drosophila melanogaster*.. Genetics.

[14] Arunyawat U, Stephan W, Städler T (2007). Using multilocus sequence data to assess population structure, natural selection, and linkage disequilibrium in wild tomatoes.. Mol Biol Evol.

[15] Roselius K, Stephan W, Städler T (2005). The relationship of nucleotide polymorphism, recombination rate and selection in wild tomato species.. Genetics.

[16] Stephan W, Langley CH (1998). DNA polymorphism in Lycopersicon and crossing-over per physical length.. Genetics.

[17] Nordborg M, Tavare S (2002). Linkage disequilibrium: what history has to tell us.. Trends Genet.

[18] Strasburg JL, Rieseberg LH (2010). How Robust Are “Isolation with Migration” Analyses to Violations of the IM Model? A Simulation Study.. Mol Biol Evol.

[19] Wakeley J, Hey J (1997). Estimating ancestral population parameters.. Genetics.

[20] Garrigan D (2009). Composite likelihood estimation of demographic parameters.. BMC Genet.

[21] Lopes JS, Balding D, Beaumont MA (2009). PopABC: a program to infer historical demographic parameters.. Bioinformatics.

[22] Gutenkunst RN, Hernandez RD, Williamson SH, Bustamante CD (2009). Inferring the Joint Demographic History of Multiple Populations from Multidimensional SNP Frequency Data.. PLoS Genet.

[23] Städler T, Arunyawat U, Stephan W (2008). Population genetics of speciation in two closely related wild tomatoes (Solanum section Lycopersicon).. Genetics.

[24] Tellier A, Fischer I, Merino C, Xia H, Camus-Kulandaivelu L (2011). Fitness effects of derived deleterious mutations in four closely related wild tomato species with spatial structure..

[25] Kimura M (1969). The number of heterozygous nucleotide sites maintained in a finite population due to steady flux of mutations.. Genetics.

[26] Hudson RR (2002). Generating samples under a Wright-Fisher neutral model of genetic variation.. Bioinformatics.

[27] Felsenstein J (1988). Phylogenies from molecular sequences: inference and reliability.. Annu Rev Genet.

[28] R Development Core Team (2005). R: A language and environment for statistical computing.

[29] McCullagh P, Nelder JA (1989). Generalized linear models 2nd edition.

[30] Karlis D, Meligkotsidou L (2005). Multivariate Poisson regression with covariance structure.. Stat Comp.

[31] Becquet C, Przeworski M (2009). Learning about modes of speciation by computational approaches.. Evolution.

[32] Thornton KR (2009). Automating approximate Bayesian computation by local linear regression.. BMC Genet.

[33] Joyce P, Marjoram P (2008). Approximately sufficient statistics and Bayesian computation.. Stat Appl Genet Mol Biol.

[34] Nunes MA, Balding DJ (2010). On optimal selection of summary statistics for Approximate Bayesian Computation.. Stat Appl Genet Mol Biol.

[35] Cornuet JM, Santos F, Beaumont MA, Robert CP, Marin JM (2008). Inferring population history with DIY ABC: a user-friendly approach to approximate Bayesian computation.. Bioinformatics.

[36] Jensen JD, Thornton KR, Aquadro CF (2008). Inferring selection in partially sequenced regions.. Mol Biol Evol.

[37] Pawitan Y (2001). In all likelihood: Statistical modelling and inference using likelihood.

[38] Efron B (1985). Bootstrap confidence intervals for a class of parametric problems.. Biometrika.

