# Supplementary material for: Estimating Parameters of Speciation Models Based on Refined Summaries of the Joint Site-Frequency Spectrum
Source: PLoS One. 2011 May 26;6(5):e18155. doi: 10.1371/journal.pone.0018155 (PMC3102651; doi:10.1371/journal.pone.0018155)
Supplement: Appendix S1 — Supplementary information. (PDF) [file pone.0018155.s001.pdf]

Appendix S1: Supplementary Informations

Content

Section 1: Four classes of the Joint-Frequency Spectrum for the Wakeley-Hey model.

Section 2: Summaries of the Joint-Frequency Spectrum for the Maximum-Likelihood method

Section 3: Summaries of the Joint-Frequency Spectrum for the Composite likelihoods methods

Section 4: Results of regression between error in estimates of divergence times and error in migration rates depending on the method.

Section 5: Analysis of variance for error in estimates of divergence times and error in migration rates depending on the method and other parameters.

Section 6: Results of the 100 datasets analysis: Factor 2, error in estimates of divergence times and errors in migration rates depending on the method and parameters.

Section 1: Four classes of the Joint-Frequency Spectrum for the Wakeley-Hey model.

The Joint site-Frequency Spectrum (JSFS) compares SNP data from n_1 samples from population 1 to n_2 samples from population 2. The JSFS is calculated as an array of dimension $(n_1 +1) \times (n_2 +1) - 2$. A cell at row i and column j contains the number of polymorphic sites $S_{i,j}$ which are found i times in population 1 and j times in population 2. For example, $S_{2,3} = 10$ if 10 polymorphisms are found as doubletons in population 1 and tripletons in population 2. Four summary statistics are relevant for isolation-migration parameter inference: private polymorphisms in species 1 and 2, respectively (W_1, W_2), fixed differences between species (W_3), and shared ancestral polymorphisms (W_4) (Wakeley and Hey 1997, see reference 19 in text).

$$W_1 = \sum_{1 \le i \le n_1 - 1} S_{i,0} + S_{i,n_2} \; ; \; W_2 = \sum_{1 \le j \le n_2 - 1} S_{0,j} + S_{n_1,j} \; ; \; W_3 = S_{0,n_2} + S_{n_1,0} \; ; \; W_4 = \sum_{1 \le i \le n_1 - 1} \sum_{1 \le j \le n_2 - 1} S_{i,j}$$

We represent the JSFS graphically, as well as the four different classes $W_{1\text{-}4}$ as follows:

	0	1	2	3	…	n_2-3	n_2-2	n_2-1	n_2
0	X				W_2				W_3
1									
3									
…	W_1				W_4				W_1
n_1-3									
n_1-2									
n_1-1									
n_1	W_3				W_2				X

<u>Section 2: Summaries of the Joint-Frequency Spectrum for the Maximum-Likelihood method</u>

We have tested four different sets of summary statistics derived from the JSFS. The four vectors of summary statistics are described below as D, D', D'', D^*.

Formally, the 7 values of vector *D* are written as:

$$D_1 = \sum_{1 \le i \le n_1 - 1} S_{i,0} \; ; \; D_2 = \sum_{1 \le j \le n_2 - 1} S_{0,j} \; ;$$

$$D_3 = S_{0,n_2} \; ; \; D_4 = S_{n_1,0} \; ;$$

$$D_5 = \sum_{1 \le i \le n_1 - 1} \sum_{1 \le j \le n_2 - 1} S_{i,j} \; ;$$

$$D_6 = \sum_{1 \le i \le n_1 - 1} S_{i,n_2} \; ; \; D_7 = \sum_{1 \le j \le n_2 - 1} S_{n_1,j}$$

In relation to Eq. 2, $W_1=D_1+D_6$, $W_2=D_2+D_7$, $W_3=D_3+D_4$ and $W_4=D_5$. In other words, $W_1=D_1+D_6$ means that we separate polymorphic SNPs in population 1 which are not found in population 2 from those that are fixed in population 2 (i.e. polarizing the private polymorphism using information from an outgroup). As above, we represent graphically the JSFS and the classes of vector *D* as follows:

<table>
<tr><th></th><th>0</th><th>1</th><th>2</th><th>3</th><th>…</th><th>n_2-3</th><th>n_2-2</th><th>n_2-1</th><th>n_2</th></tr>
<tr><td>0</td><td>X</td><td colspan="8">D_2</td><td>D_3</td></tr>
<tr><td>1</td><td rowspan="7">D_1</td><td rowspan="7" colspan="8">D_5</td><td rowspan="7">D_6</td></tr>
<tr><td>2</td></tr>
<tr><td>3</td></tr>
<tr><td>…</td></tr>
<tr><td>n_1-3</td></tr>
<tr><td>n_1-2</td></tr>
<tr><td>n_1-1</td></tr>
<tr><td>n_1</td><td>D_4</td><td colspan="8">D_7</td><td>X</td></tr>
</table>

The second decomposition D_k' (k=1,..,12) is based on extracting the number of singletons from various classes of D.

$$D'_1 = S_{0,1}\,;\; D'_2 = \sum_{2\le i\le n_1-1} S_{i,0}\,;\; D'_3 = S_{1,0}\,;\; D'_4 = \sum_{2\le j\le n_2-1} S_{0,j}\,;$$

$$D'_5 = S_{0,n_2}\,;\; D'_6 = S_{n_1,0}\,;\; D'_7 = S_{1,1}\,;\; D'_8 = S_{1,n_2}\,;\; D'_9 = S_{n_1,1}$$

$$D'_{10} = \sum_{2\le i\le n_1-1}\sum_{2\le j\le n_2-1} S_{i,j}\,;\; D'_{11} = \sum_{2\le i\le n_1-1} S_{i,n_2}\,;\; D'_{12} = \sum_{2\le j\le n_2-1} S_{n_1,j}$$

	0	1	2	3	…	n_2-3	n_2-2	n_2-1	n_2
0	X	***D'***$_1$			***D'***$_4$				***D'***$_5$
1	***D'***$_3$	***D'***$_7$							***D'***$_8$
2									
3									
…	***D'***$_2$				***D'***$_{10}$				***D'***$_{11}$
n_1-3									
n_1-2									
n_1-1									
n_1	***D'***$_6$	***D'***$_9$			***D'***$_{12}$				X

The third decomposition D_k''(k=1,..,12) contains low frequency polymorphism defined by singletons and doubletons from various classes of D.

$$D''_1 = S_{0,1} + S_{0,2}\,;\; D''_2 = \sum_{3 \le i \le n_1 - 1} S_{i,0}\,;\; D''_3 = S_{1,0} + S_{2,0}\,;\; D''_4 = \sum_{3 \le j \le n_2 - 1} S_{0,j}\,;$$

$$D''_5 = S_{0,n_2}\,;\; D''_6 = S_{n_1,0}\,;\; D''_7 = S_{1,1} + S_{1,2} + S_{2,1} + S_{2,2}\,;\; D''_8 = S_{1,n_2} + S_{2,n_2}\,;\; D''_9 = S_{n_1,1} + S_{n_1,2}$$

$$D''_{10} = \sum_{3 \le i \le n_1 - 1} \sum_{3 \le j \le n_2 - 1} S_{i,j}\,;\; D''_{11} = \sum_{3 \le i \le n_1 - 1} S_{i,n_2}\,;\; D''_{12} = \sum_{3 \le j \le n_2 - 1} S_{n_1,j}$$

	0	1	2	3	…	n_2-3	n_2-2	n_2-1	n_2
0	X	***D''$_1$***		***D''$_4$***					***D''$_5$***
1	***D''$_3$***	***D''$_7$***							***D''$_8$***
2									
3	***D''$_2$***								***D''$_{12}$***
…					***D''$_{10}$***				
n_1-3									
n_1-2									
n_1-1									
n_1	***D''$_6$***	***D''$_9$***		***D''$_{11}$***					X

The fourth decomposition, the vector D_k^* (k=1,…,23), countains singletons and doubletons in separate classes.

$$D_1^* = S_{0,1}\,;\; D_2^* = S_{0,2}\,;\; D_3^* = \sum_{3\le j\le n_2-1} S_{0,j}\,;\; D_4^* = \sum_{3\le i\le n_1-1} S_{i,0}\,;$$

$$D_5^* = S_{1,0}\,;\; D_6^* = S_{1,1}\,;\; D_7^* = S_{1,2}\,;\; D_8^* = S_{2,0}\,;\; D_9^* = S_{2,1}\,;\; D_{10}^* = S_{2,2}\,;$$

$$D_{11}^* = S_{0,n_2}\,;\; D_{12}^* = S_{1,n_2}\,;\; D_{13}^* = S_{2,n_2}\;\; D_{14}^* = S_{n_1,0}\,;\; D_{15}^* = S_{n_1,1}\,;\; D_{16}^* = S_{n_1,2}\,;$$

$$D_{17}^* = \sum_{3\le j\le n_2-1} S_{1,j}\,;\; D_{18}^* = \sum_{3\le j\le n_2-1} S_{2,j}\,;\; D_{19}^* = \sum_{3\le i\le n_1-1} S_{i,1}\,;\; D_{20}^* = \sum_{3\le i\le n_1-1} S_{i,2}\,;$$

$$D_{21}^* = \sum_{3\le i\le n_1-1}\sum_{3\le j\le n_2-1} S_{i,j}\,;\; D_{22}^* = \sum_{3\le i\le n_1-1} S_{i,n_2}\,;\; D_{23}^* = \sum_{3\le j\le n_2-1} S_{n_1,j}$$

<table>
<tr><th></th><th>0</th><th>1</th><th>2</th><th>3</th><th>…</th><th>n2-3</th><th>n2-2</th><th>n2-1</th><th>n2</th></tr>
<tr><td>0</td><td>X</td><td>D*1</td><td>D*2</td><td colspan="5">D*3</td><td>D*11</td></tr>
<tr><td>1</td><td>D*5</td><td>D*6</td><td>D*7</td><td colspan="5">D*17</td><td>D*12</td></tr>
<tr><td>2</td><td>D*8</td><td>D*9</td><td>D*10</td><td colspan="5">D*18</td><td>D*13</td></tr>
<tr><td>3</td><td rowspan="5">D*4</td><td rowspan="5">D*19</td><td rowspan="5">D*20</td><td colspan="5" rowspan="5">D*21</td><td rowspan="5">D*22</td></tr>
<tr><td>…</td></tr>
<tr><td>n1-3</td></tr>
<tr><td>n1-2</td></tr>
<tr><td>n1-1</td></tr>
<tr><td>n1</td><td>D*14</td><td>D*15</td><td>D*16</td><td colspan="5">D*23</td><td>X</td></tr>
</table>

Section 3: Summaries of the Joint-Frequency Spectrum for the Composite-Likelihood analysis method

Here we consider singletons, doubletons, and polymorphic sites with high frequencies n_1 -1 and n_1 -2 in population 1 or n_2 -1 and n_2 -2 in population 2 ($\breve{D}_k$, k=1,…,23) separately.

$$\breve{D}_1 = S_{1,0} + S_{2,0}\,;\ \breve{D}_2 = \sum_{3\le i\le n_1-3} S_{i,0}\,;\ \breve{D}_3 = S_{n_1-2,0} + S_{n_1-1,0}\,;\ \breve{D}_4 = S_{n_1,0}\,;$$

$$\breve{D}_5 = S_{0,1} + S_{0,2}\,;\ \breve{D}_6 = S_{1,1} + S_{2,1} + S_{1,2} + S_{2,2}\,;\ \breve{D}_7 = \sum_{3\le i\le n_1-3} S_{i,1} + \sum_{3\le i\le n_1-3} S_{i,2}\,;$$

$$\breve{D}_8 = S_{n_1-2,1} + S_{n_1-1,1} + S_{n_1-2,2} + S_{n_1-1,2}\,;\ \breve{D}_9 = S_{n_1,1} + S_{n_1,2}\,;\ \breve{D}_{10} = \sum_{3\le j\le n_2-3} S_{0,j}\,;$$

$$\breve{D}_{11} = \sum_{3\le j\le n_2-3} S_{1,j} + \sum_{3\le j\le n_2-3} S_{2,j}\,;\ \breve{D}_{12} = \sum_{3\le i\le n_1-3}\ \sum_{3\le j\le n_2-3} S_{i,j}\,;\ \breve{D}_{13} = \sum_{3\le j\le n_2-3} S_{n_1-2,j} + \sum_{3\le j\le n_2-3} S_{n_1-1,j}\,;$$

$$\breve{D}_{14} = \sum_{3\le j\le n_2-3} S_{n_1,j}\,;\ \breve{D}_{15} = S_{0,n_2-2} + S_{0,n_2-1}\,;\ \breve{D}_{16} = S_{1,n_2-2} + S_{1,n_2-1} + S_{2,n_2-2} + S_{2,n_2-1}\,;$$

$$\breve{D}_{17} = \sum_{3\le i\le n_1-3} S_{i,n_2-2} + \sum_{3\le i\le n_1-3} S_{i,n_2-1}\,;\ \breve{D}_{18} = S_{n_1-2,n_2-2} + S_{n_1-2,n_2-1} + S_{n_1-1,n_2-2} + S_{n_1-1,n_2-1}\,;$$

$$\breve{D}_{19} = S_{n_1,n_2-2} + S_{n_1,n_2-1}\,;\ \breve{D}_{20} = S_{0,n_2}\,;\ \breve{D}_{21} = S_{1,n_2} + S_{2,n_2}\,;\ \breve{D}_{22} = \sum_{3\le i\le n_1-3} S_{i,n_2}\,;\ \breve{D}_{23} = S_{n_1-2,n_2} + S_{n_1-1,n_2}$$

For a simple model with equal mutation rates in the two populations ($\theta_1 = \theta_2$) and equal gene flow rates ($M_{12} = M_{21}$), we verify by coalescent simulations of the Wakeley-Hey model that the JSFS shows an axis of symmetry along the diagonal (0,0) to (n_1,n_2). In this case, symmetry also appears among elements of the *J* vector, namely: $\check{D}_1 = \check{D}_5$, $\check{D}_2 = \check{D}_{10}$, $\check{D}_3 = \check{D}_{15}$, $\check{D}_4 = \check{D}_{20}$, $\check{D}_7 = \check{D}_{11}$, $\check{D}_8 = \check{D}_{16}$, $\check{D}_9 = \check{D}_{21}$, $\check{D}_{13} = \check{D}_{17}$, $\check{D}_{14} = \check{D}_{22}$, $\check{D}_{19} = \check{D}_{23}$. This means that the number of sites with a mutation fixed in population 1 and absent in population 2 ($\check{D}_4$) is equal to the number of mutations fixed in population 2 and absent in population 1 ($\check{D}_{20}$).

<table>
<tr><td></td><td>0</td><td>1</td><td>2</td><td>3</td><td>…</td><td>n_2-3</td><td>n_2-2</td><td>n_2-1</td><td>n_2</td></tr>
<tr><td>0</td><td>X</td><td colspan="2">$\check{D}_5$</td><td colspan="3">$\check{D}_{10}$</td><td colspan="2">$\check{D}_{15}$</td><td>$\check{D}_{20}$</td></tr>
<tr><td>1</td><td rowspan="2">$\check{D}_1$</td><td colspan="2" rowspan="2">$\check{D}_6$</td><td colspan="3" rowspan="2">$\check{D}_{11}$</td><td colspan="2" rowspan="2">$\check{D}_{16}$</td><td rowspan="2">$\check{D}_{21}$</td></tr>
<tr><td>2</td></tr>
<tr><td>3</td><td rowspan="3">$\check{D}_2$</td><td colspan="2" rowspan="3">$\check{D}_7$</td><td colspan="3" rowspan="3">$\check{D}_{12}$</td><td colspan="2" rowspan="3">$\check{D}_{17}$</td><td rowspan="3">$\check{D}_{22}$</td></tr>
<tr><td>…</td></tr>
<tr><td>n_1-3</td></tr>
<tr><td>n_1-2</td><td rowspan="2">$\check{D}_3$</td><td colspan="2" rowspan="2">$\check{D}_8$</td><td colspan="3" rowspan="2">$\check{D}_{13}$</td><td colspan="2" rowspan="2">$\check{D}_{18}$</td><td rowspan="2">$\check{D}_{23}$</td></tr>
<tr><td>n_1-1</td></tr>
<tr><td>n_1</td><td>$\check{D}_4$</td><td colspan="2">$\check{D}_9$</td><td colspan="3">$\check{D}_{14}$</td><td colspan="2">$\check{D}_{19}$</td><td>X</td></tr>
</table>

Section 4: Relative of error in estimates of divergence times and error in migration rates depending on the method.

Figure S1a and b present the results of the power analysis for sets of 7 loci with 20 replicates (results in text in Figures 1-2), for given values of θ and ρ. Note that method J_1 has 8 possible estimated values (the means of each block) for τ and M. In these datasets where $\tau = 0.1$, only three values for the divergence time were estimated. The value of $\tau = 0.13954$ (relative error = 0.39541, black rectangle in Figure S1a) was the most frequently estimated value with 111 occurrences over the 140 datasets using method J_1.

In Figure S2, for all nine methods, positive correlations are found between the relative bias in estimates of divergence time and migration rates. This means that when a method over (under)-estimates the divergence time, it also over (under)-estimates the migration rate.

Section 5: Analysis of variance for error in estimates of divergence times and error in migration rates depending on the method and other parameters.

The analysis of variance was performed using the *glm* function, and multiple mean comparisons are based on Tukey's HSD test (confirmed by Bonferroni test) as implemented in the R software (R DEVELOPMENT CORE TEAM 2005). Groups of significance for the multiple comparison tests are shown on Figure S1a. In the *glm* function we use the option: family = Gaussian. We considered all possible two way and three way interaction terms between the different parameters (Method, θ, ρ, M), and sequentially remove non-significant interactions. P-values for single parameters and the interaction term Method*θ are similar to those of Table S1 when only those four terms are considered in the ANOVA formula. Here we show the significance (or non-significance) of interesting interactions for the behavior of the different methods (Method= D_1-D_4, MIMAR, J_1-J_4).

The analysis of variance was performed using the *glm* function, and multiple mean comparisons are based on Tukey's HSD test (confirmed by Bonferroni test) as implemented in the R software (R DEVELOPMENT CORE TEAM 2005). Groups of significance for the multiple comparison tests are shown on Figure S1b. In the *glm* function we use the option: family = Gaussian.

We considered all possible two way and three way interaction terms between the different parameters (Method, θ, ρ, M), and sequentially removed non-significant interactions. P-values for single parameters and the interaction term θ*M are similar to those of Table S2 when only those four terms are considered in the ANOVA formula. Here we show the significance (or non-significance) of interesting interactions for the behavior of the different methods (Method= D_1-D_4, MIMAR, J_1-J_4).

Section 6: Results of the 100 datasets analysis: Factor 2, error in estimates of divergence times and errors in migration rates depending on the method and other parameters.

Figures S6 and S7 highlight the absence of any clear correlation between error in estimating WH parameters and the population size (θ) or the recombination rate (ρ). These conclusions are valid for all composite-likelihood methods and popABC results.

Figures S7 and S8 show that estimates of migration rates are less accurate for recent divergence times (τ<0.5; difference in scale of the y-axes in Figures S8a and S9a, S8b and S9b). Moreover, with composite methods J_2 and J_4, high migration rates can be better estimated (have little relative error) even with recent divergence (<0.5; Figures S8a and S9a). However, we do not find the same trend for popABC (Figure S10), showing the inaccuracy of estimating migration rates with this method independent of divergence.
