# Supplementary figures and images for: Estimating Parameters of Speciation Models Based on Refined Summaries of the Joint Site-Frequency Spectrum
Source: PLoS One. 2011 May 26;6(5):e18155. doi: 10.1371/journal.pone.0018155 (PMC3102651; doi:10.1371/journal.pone.0018155)

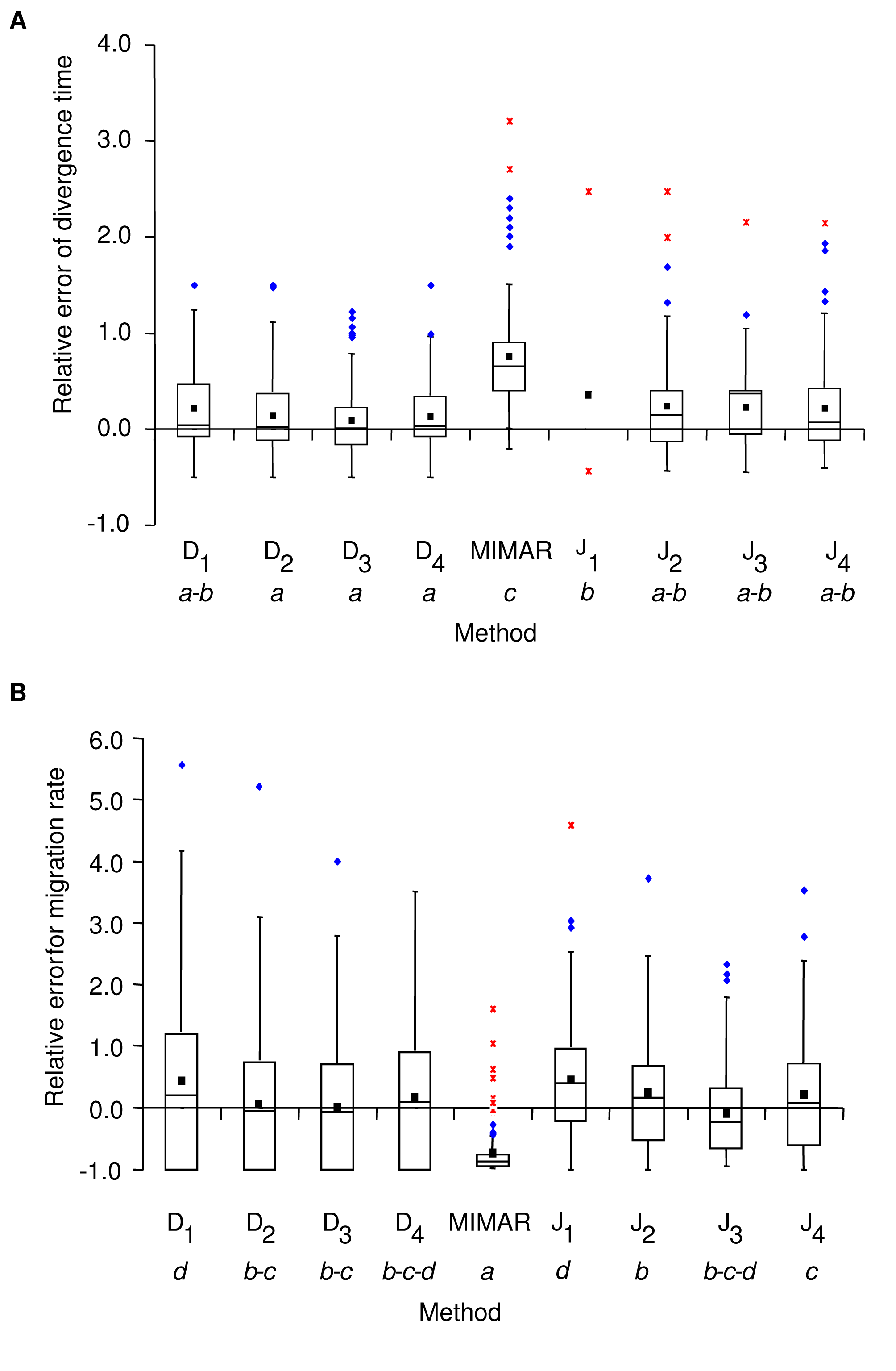

Supplement: Figure S1 — Relative error for estimates of (a) the divergence time ( τ ) and (b) the migration rate ( M = M12 = M21 ), for the maximum likelihood methods (D1–D4), MIMAR and the composite-likelihood methods (J1–J4). Relative error is calculated as (τest−τsim)/τsim where τest is the estimated value and τsim is the simulated value. Groups with significant differences between means following multiple comparisons (Tukey HSD test at 0.05) are indicated by letters for each method (group a for the smallest mean). Values that are more than 1.5 times the nearest interquartile range (25% or 75%) are displayed as diamonds, those more than 3 times are displayed as stars. (TIF) [file pone.0018155.s002.tif]

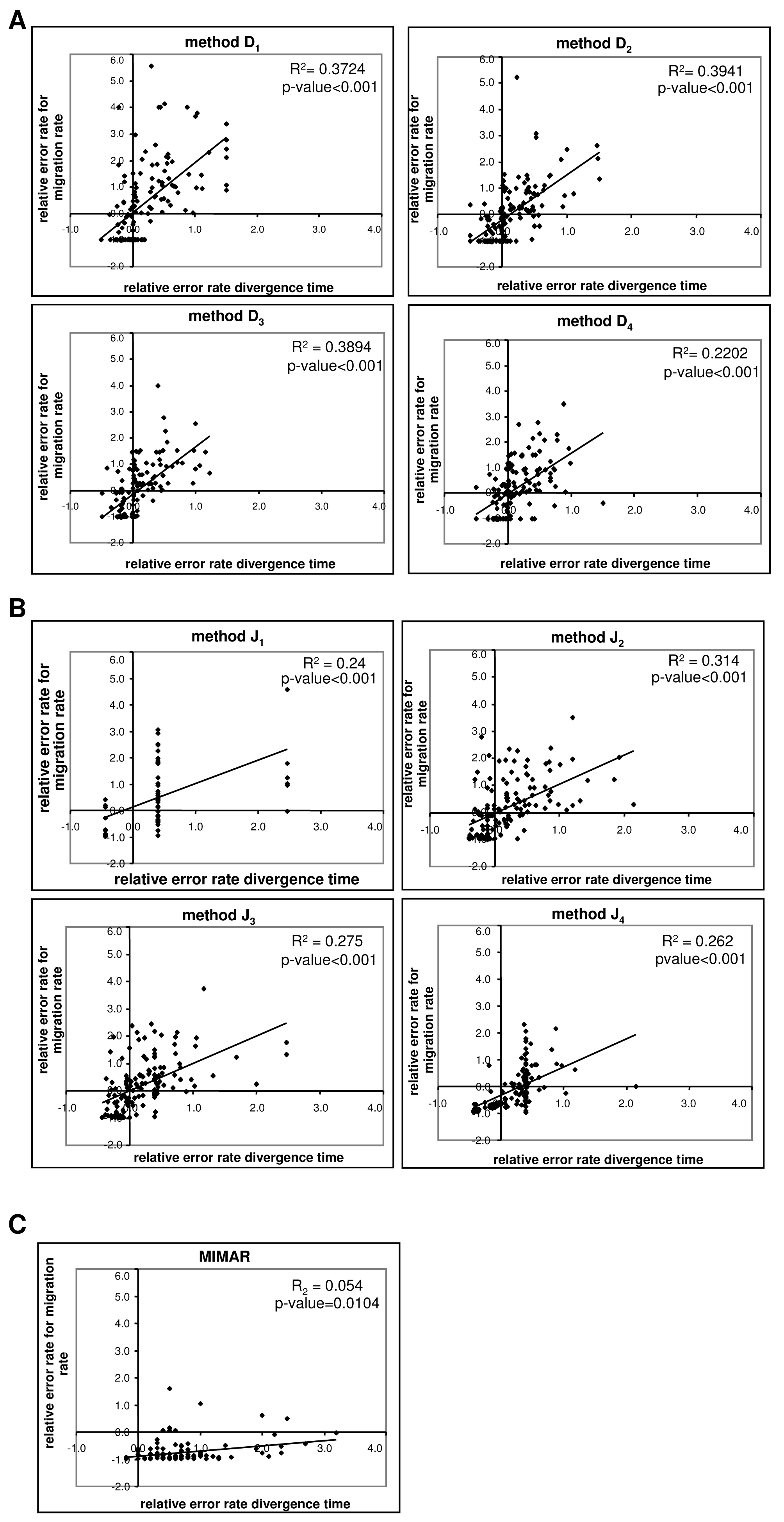

Supplement: Figure S2 — Analysis of regression between errors in estimates of migration rate ( M12 = M21 ) and divergence time τ for the 9 methods tested. (a) D1–4 for the maximum likelihood methods, (b) J1–4 for the composite likelihood methods and (c) for MIMAR. Positive (negative) relative error indicates over (under)-estimation of the parameter. Regression coefficients and p-values are calculated using the lm function in the R software. P-values indicate the significance of the test whether the slope of the linear regression is zero. (TIF) [file pone.0018155.s003.tif]

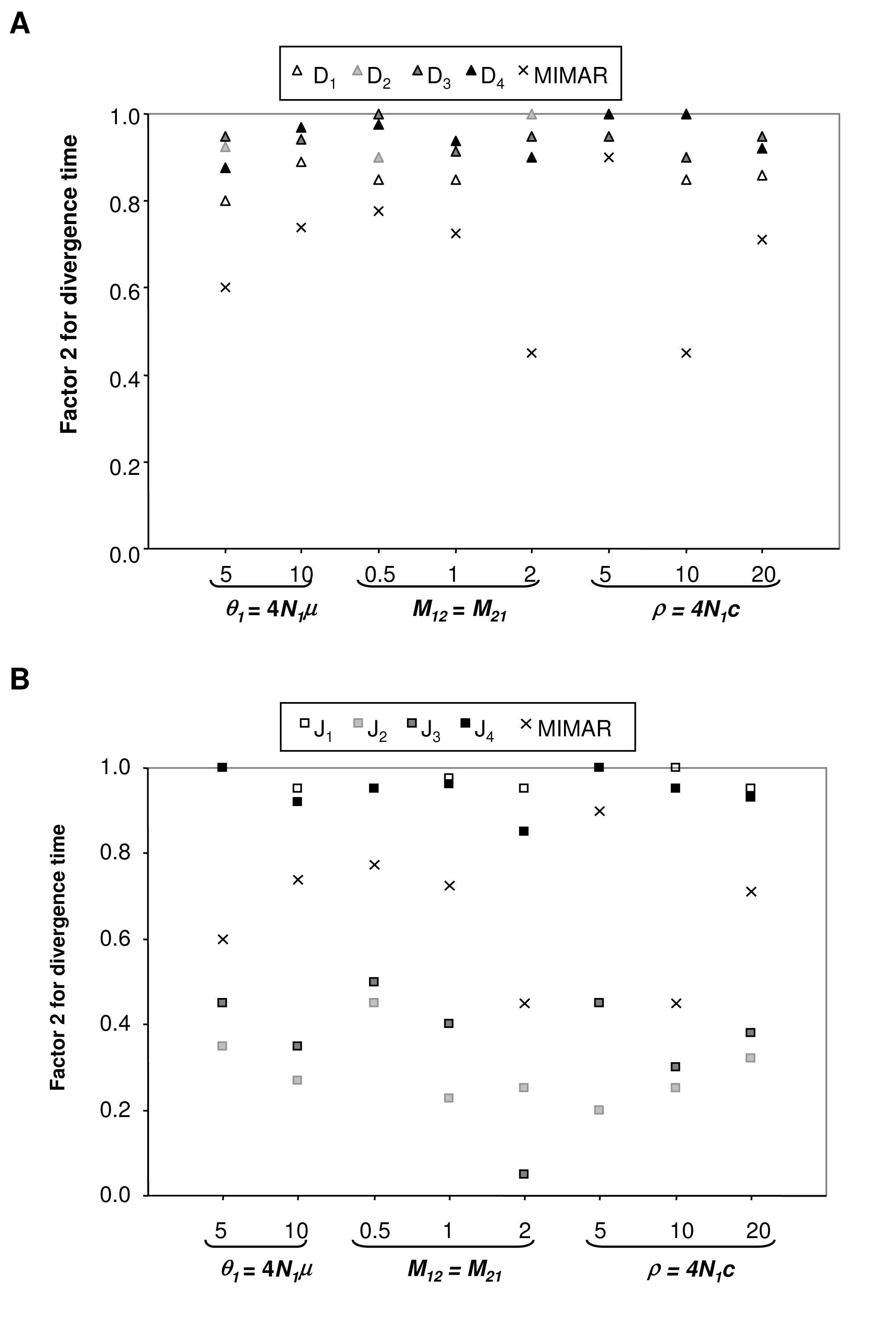

Supplement: Figure S3 — Factor 2 as a percentage of the estimates of divergence time ( τ ) in the range τsim /2< τest < τsim ×2 as a function of the population mutation rates ( θ ), values of simulated migration rates ( M12 = M21 ) and population recombination rates ( ρ ). The Factor 2 (F2) is the proportion of data sets for which the estimated value (of τ or M) is at least half and at most twice the simulated value: (a) for the four maximum likelihood methods (D1–D4) and MIMAR, (b) for the four composite-likelihood methods (J1–J4) and MIMAR. (TIF) [file pone.0018155.s004.tif]

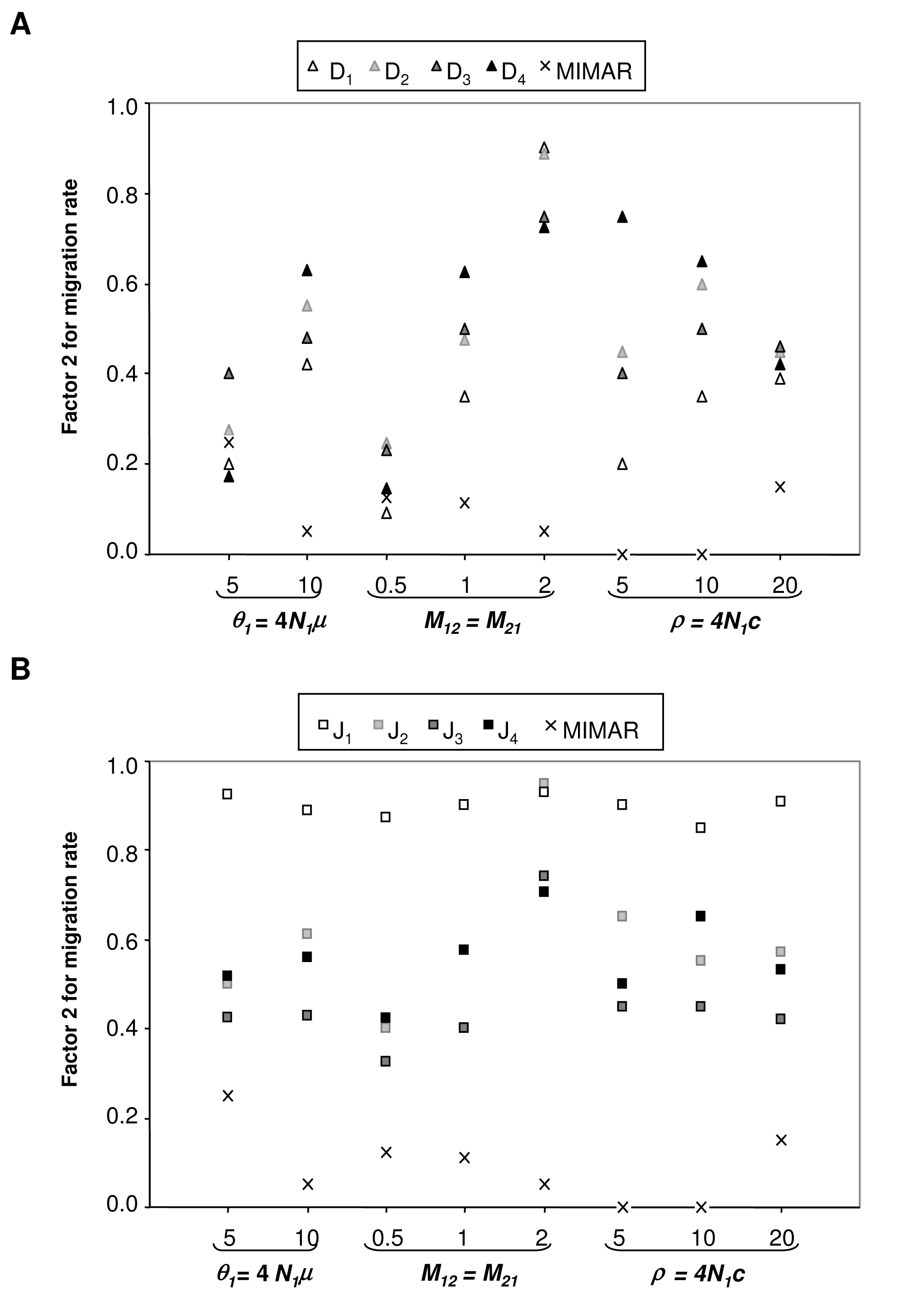

Supplement: Figure S4 — Factor 2 as a percentage of the estimates of migration rate ( M = M12 = M21 ) in the range Msim /2< Mest < Msim ×2 as a function of the population mutation rate ( θ ), values of simulated migration rates ( M12 = M21 ) and population recombination rates ( ρ ). (a) For the four maximum likelihood methods (D1–D4) and MIMAR, (b) for the four composite-likelihood methods (J1–J4) and MIMAR. (TIF) [file pone.0018155.s005.tif]

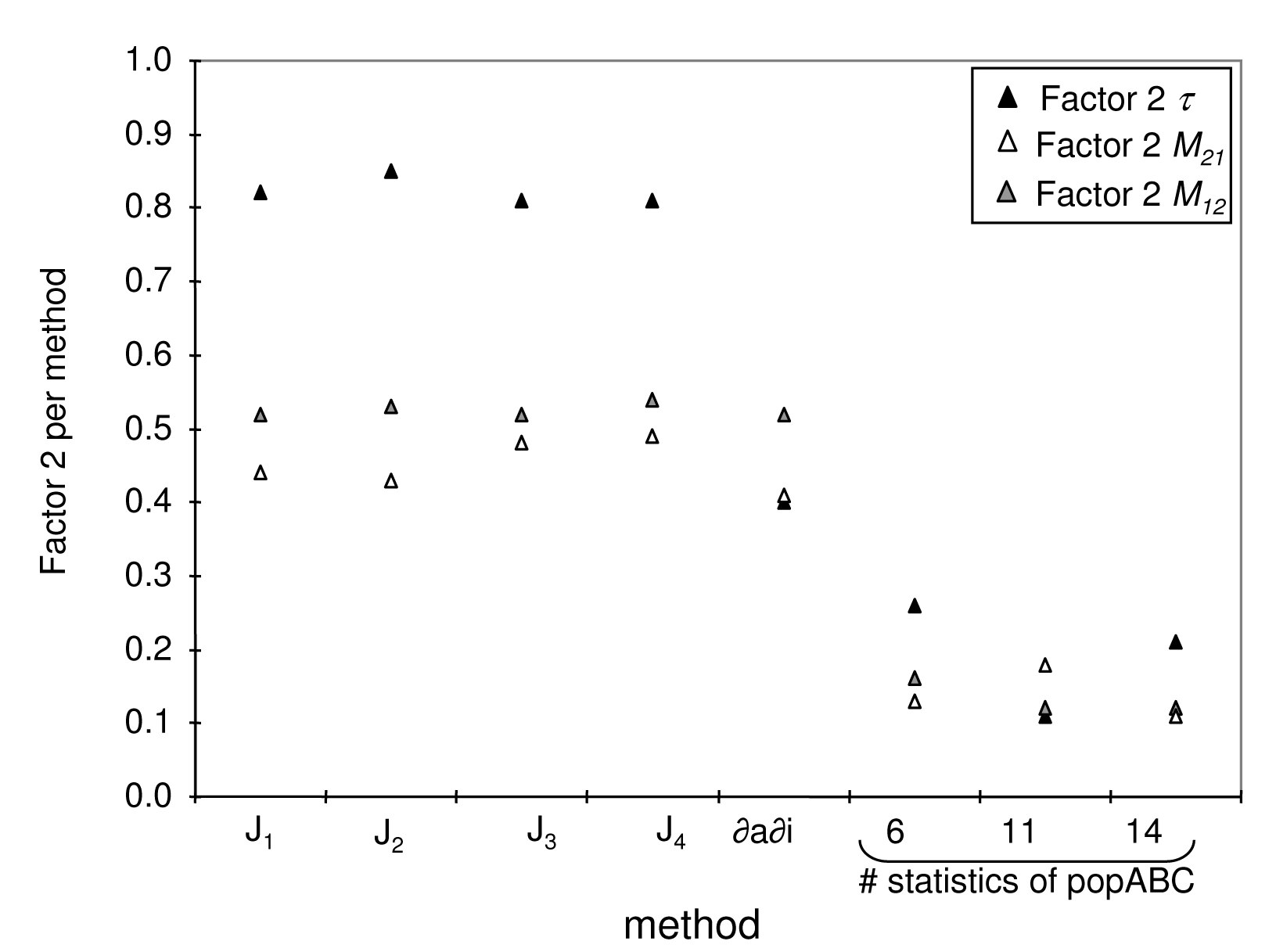

Supplement: Figure S5 — Factor 2 for estimates of the divergence time and migration rates ( M12 , M21 ) for the four composite-likelihood methods (J1–J4), ∂a∂i and for popABC with 6, 11 and 14 summary statistics (computed over 100 datasets). (TIF) [file pone.0018155.s006.tif]

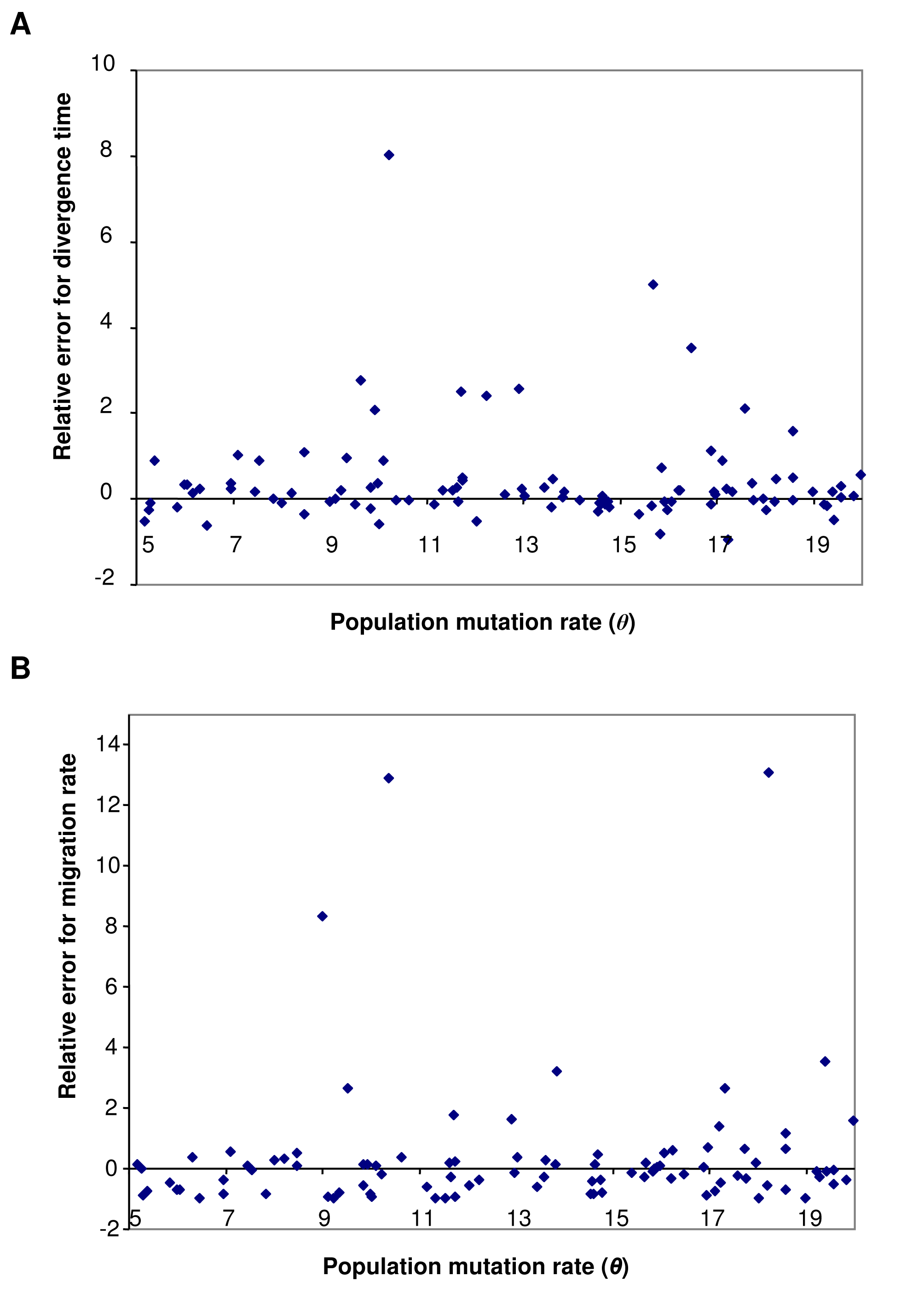

Supplement: Figure S6 — Distribution of relative error for (a) divergence time and for (b) migration rate depending on the population mutation rate ( θ ) for composite-likelihood method J4. For clarity, only relative errors lower than 15 are shown in (b). (TIF) [file pone.0018155.s007.tif]

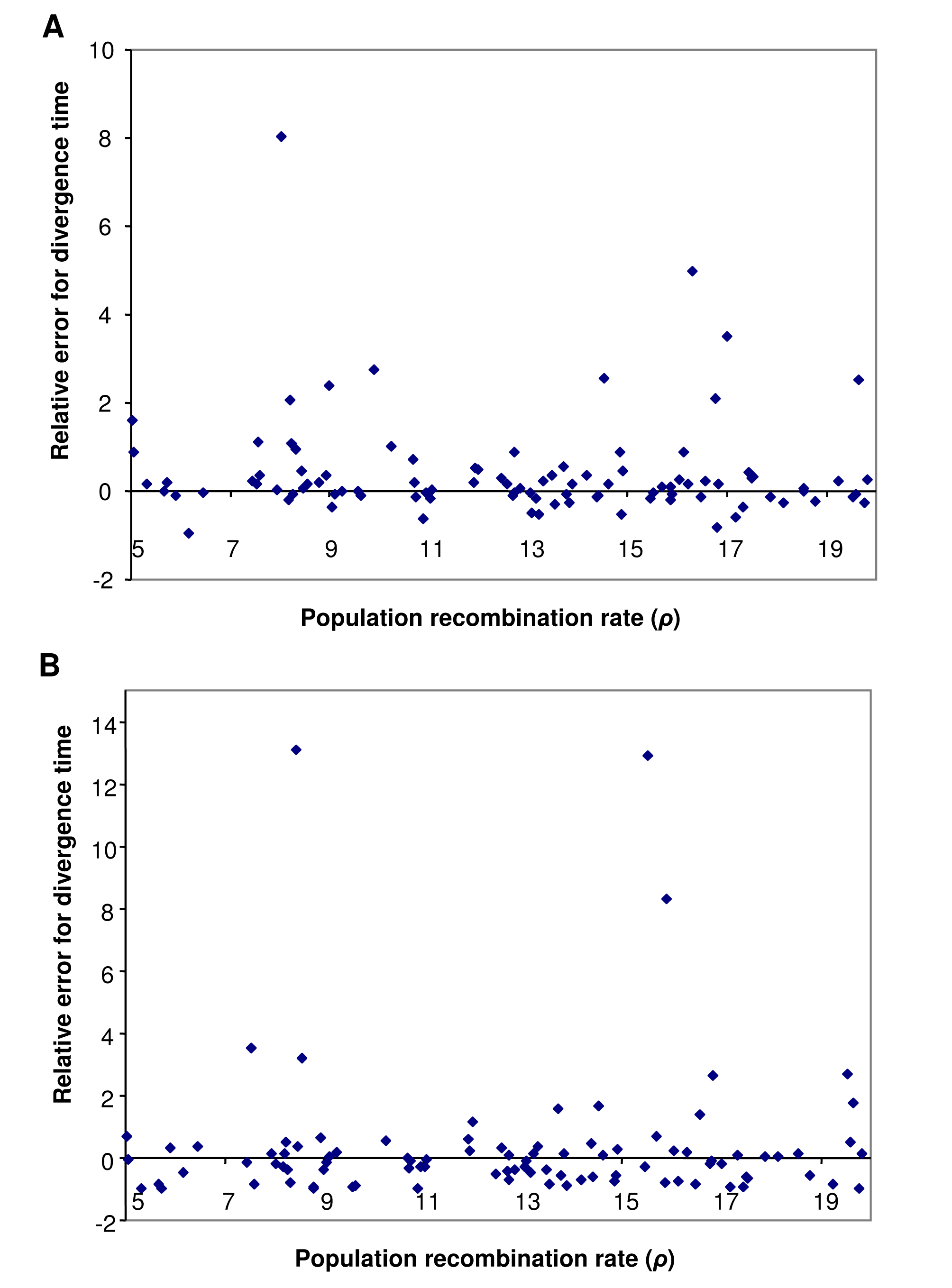

Supplement: Figure S7 — Distribution of the relative error of (a) divergence time and of (b) migration rate depending on the population recombination rate ( ρ ) for composite-likelihood method J4. For clarity, only relative errors lower than 15 are shown in (b). (TIF) [file pone.0018155.s008.tif]

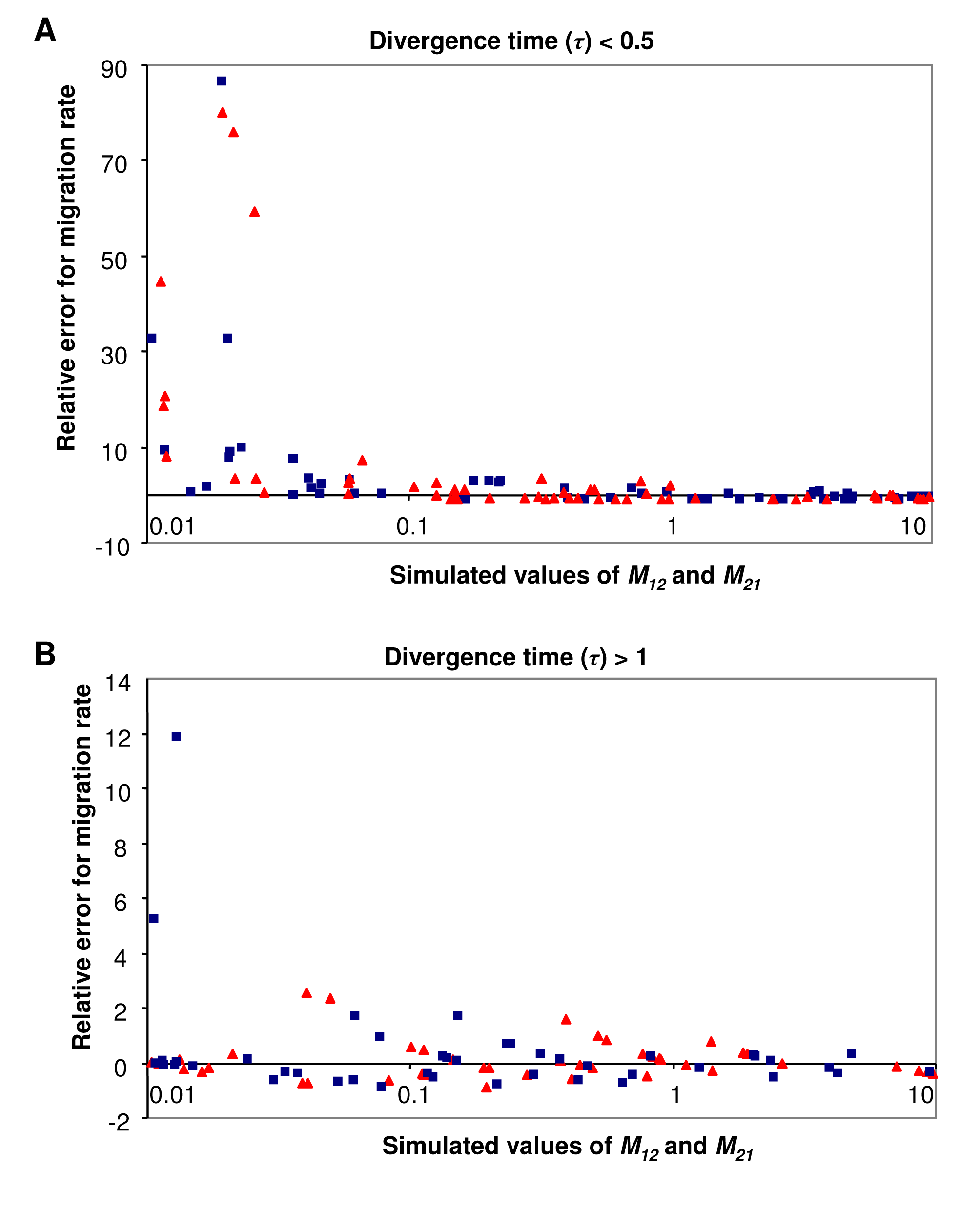

Supplement: Figure S8 — Relative error for estimation of migration rate depending on the simulated value of the migration rate ( M12 in blue and M21 in red) for composite method J2. (a) For simulated divergence times less than 0.5, and (b) for simulated divergence times greater than 1. Note the difference in scale of the y-axes between (a) and (b). (TIF) [file pone.0018155.s009.tif]

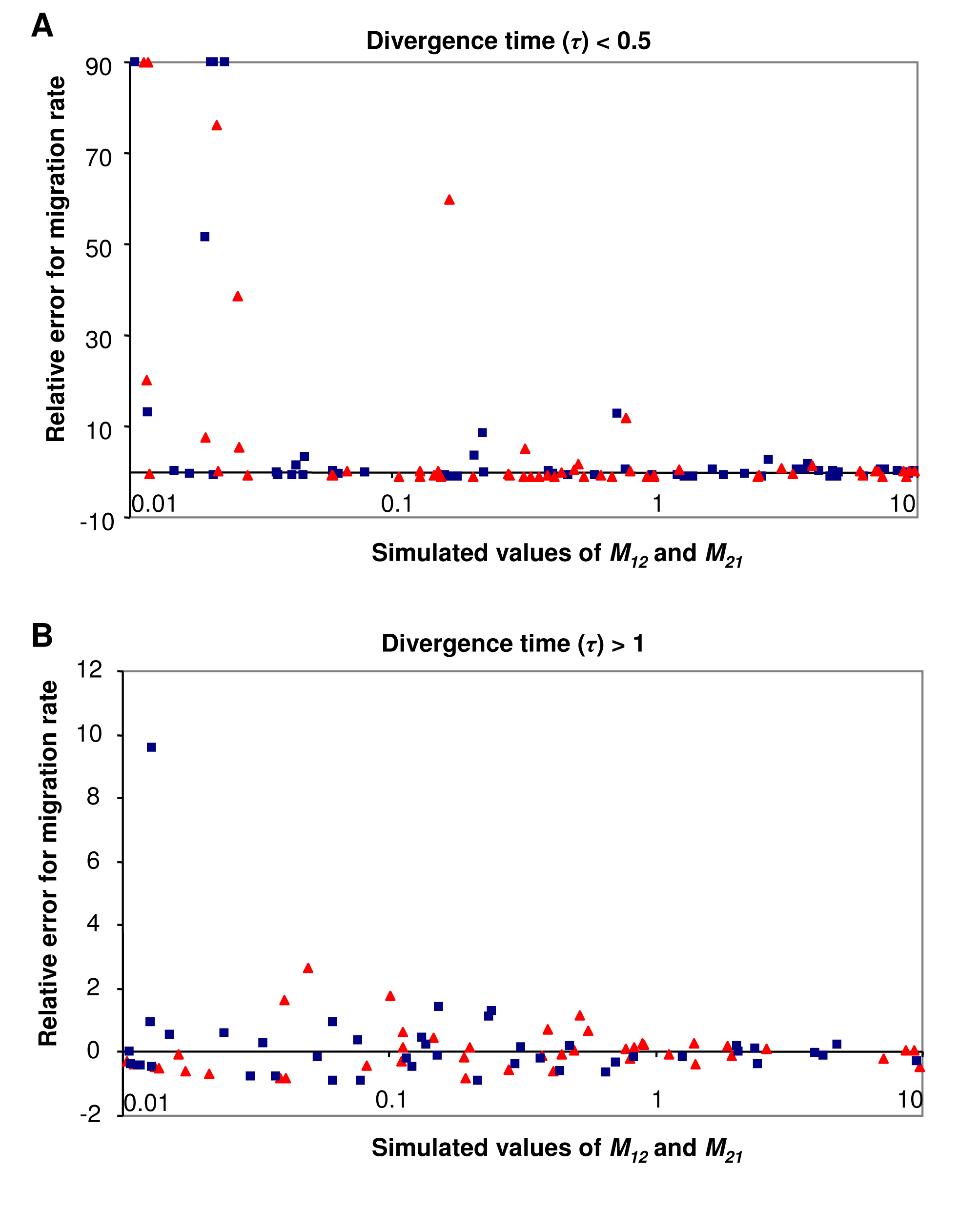

Supplement: Figure S9 — Relative error in the estimation of the migration rate ( M12 in blue and M21 in red) depending on the simulated value of the migration rate for composite likelihood method J4. (a) For simulated divergence times smaller than 0.5, and (b) for simulated divergence times greater than 1. Note the difference in scale of the y-axes between (a) and (b). (TIF) [file pone.0018155.s010.tif]

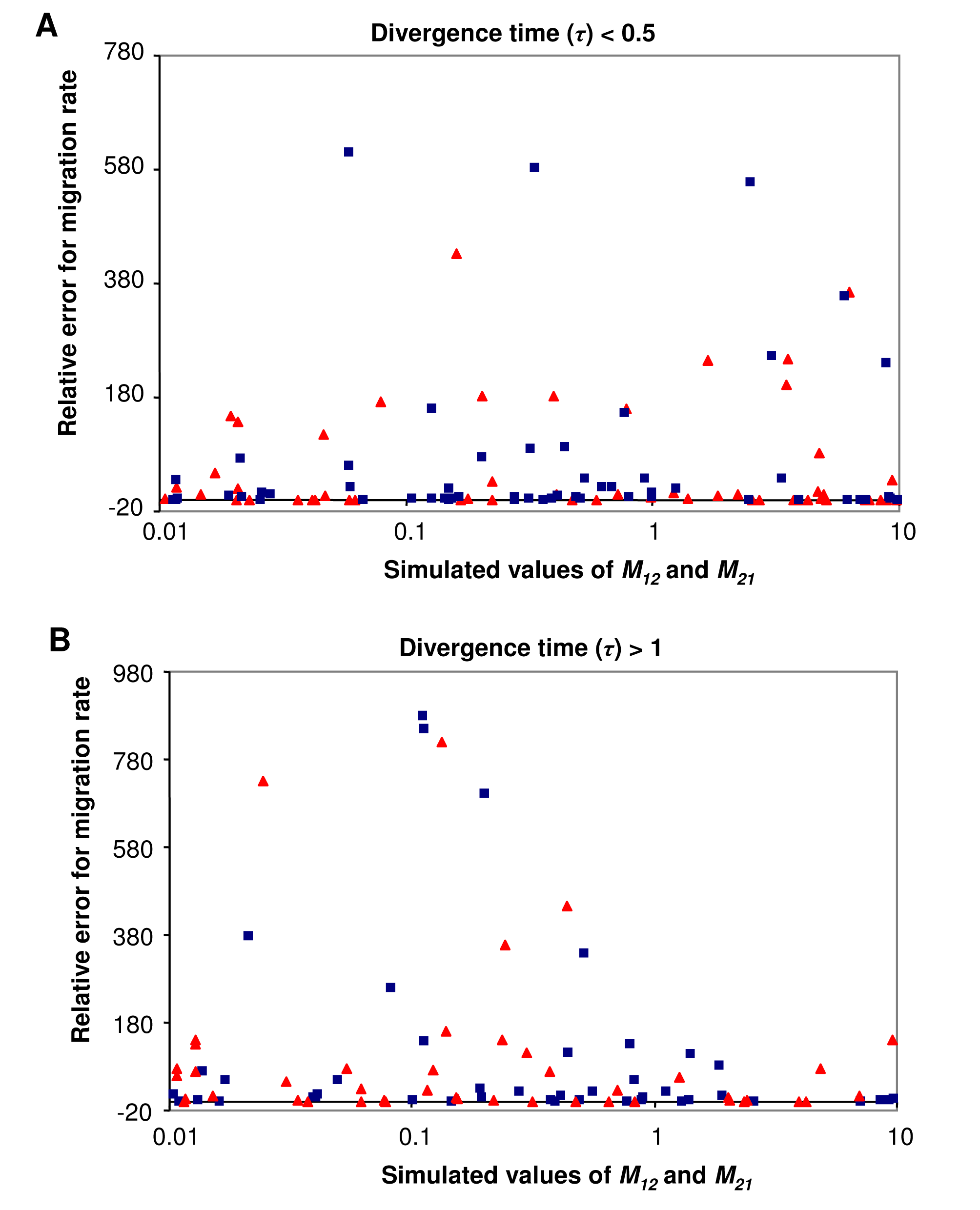

Supplement: Figure S10 — Relative error in the estimation of migration rate depending on the simulated value of the migration rate ( M12 in blue and M21 in red) for popABC estimates with 6 summary statistics. (a) For simulated divergence times smaller than 0.5, and (b) for simulated divergence times greater than 1. (TIF) [file pone.0018155.s011.tif]

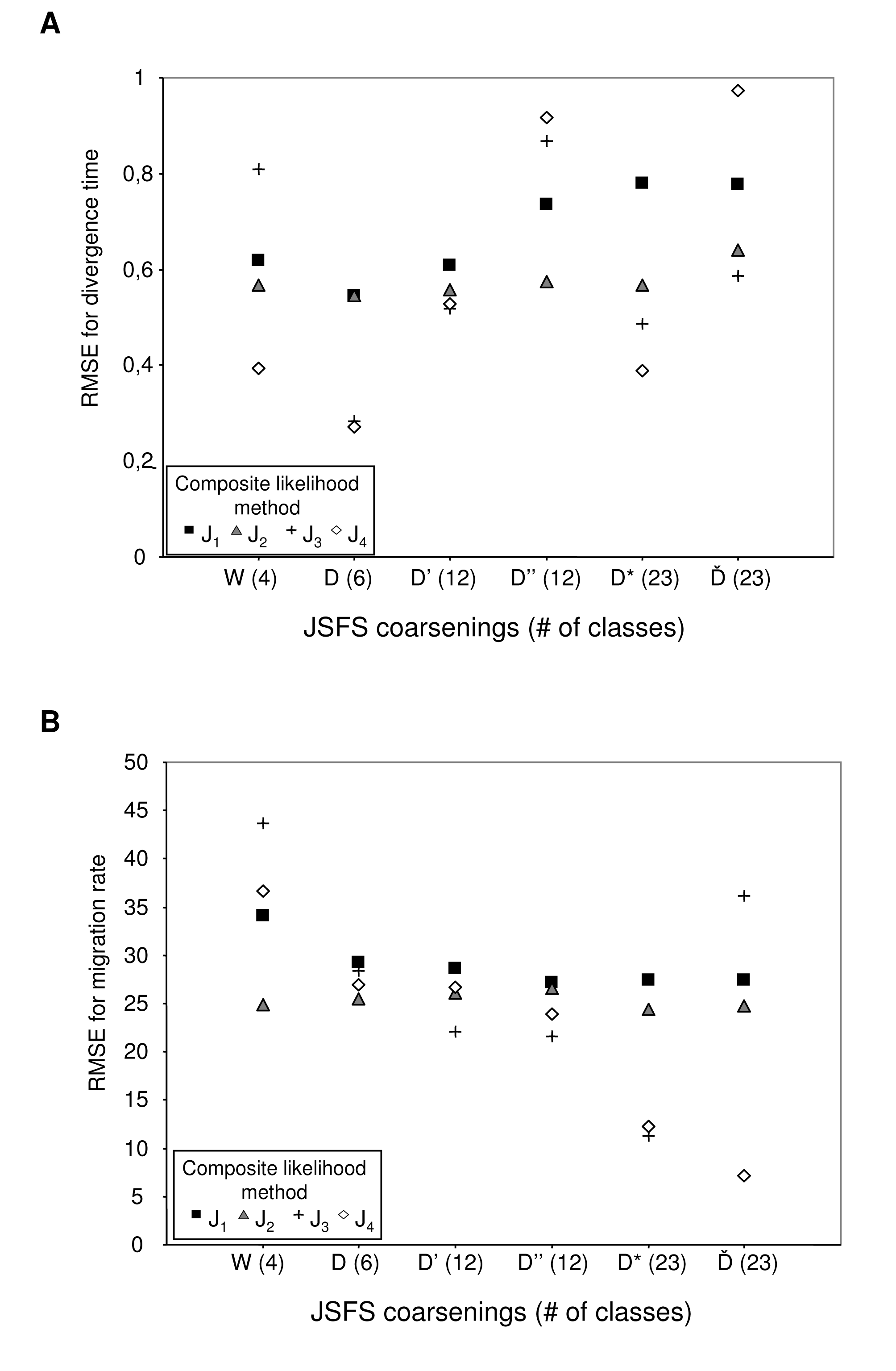

Supplement: Figure S11 — Power analysis of the various JSFS coarsenings to estimate divergence time and migration rates for 100 datasets of 100 loci. RMSE are computed for estimates of the (a) divergence time (τ) and (b) migration rates (M12≠M21) for the four composite-likelihood methods (J1–J4) based on six vectors of summary statistics with different numbers elements. The vector W is defined by the Wakeley-Hey 4 classes from Eq. 2, and other vectors D, D′, D″, D* and Ď are refined decompositions of the JSFS with higher number of classes. (TIF) [file pone.0018155.s012.tif]
