# Supplementary material for: Estimating Parameters of Speciation Models Based on Refined Summaries of the Joint Site-Frequency Spectrum
Source: PLoS One. 2011 May 26;6(5):e18155. doi: 10.1371/journal.pone.0018155 (PMC3102651; doi:10.1371/journal.pone.0018155)
Supplement: Table S1 — ANOVA table of analysis of error in the estimation of divergence times ( τ ). (PDF) [file pone.0018155.s013.pdf]

Table S1: ANOVA table of analysis of error in the estimation of divergence times (τ).

	Df	Sum of squares	Mean Square	F value	p-value
θ (population mutation rate)	1	1.897	1.897	9.4	<0.01**
ρ (recombination rate)	1	0.126	0.126	0.626	0.429
M (migration rate)	1	0.431	0.431	2.137	0.144
Method* θ	8	6.993	0.874	4.332	<0.0001***
Method* ρ	8	0.748	0.094	0.463	0.882
Method* M	8	2.656	0.332	1.645	0.107
θ * M	1	0.261	0.261	1.292	0.256
Method * θ * M	8	0.916	0.115	0.567	0.805
Residuals	1195	294.811	0.247		
