# Supplementary material for: Estimating Parameters of Speciation Models Based on Refined Summaries of the Joint Site-Frequency Spectrum
Source: PLoS One. 2011 May 26;6(5):e18155. doi: 10.1371/journal.pone.0018155 (PMC3102651; doi:10.1371/journal.pone.0018155)
Supplement: Table S2 — ANOVA table of analysis of error in the estimation of migration rates ( M12 = M21 ). (PDF) [file pone.0018155.s014.pdf]

Table S2: ANOVA table of analysis of error in the estimation of migration rates ($M_{12}=M_{21}$).

	Df	Sum of squares	Mean Square	F value	p-value
Method	8	127.58	15.948	16.093	<0.0001***
θ (population mutation rate)	1	0.54	0.54	0.540	0.463
ρ recombination rate)	1	2.18	2.18	2.205	0.138
M (migration rate)	1	10.72	10.72	10.822	<0.01**
Method*θ	8	7.40	0.925	0.934	0.487
Method* ρ	8	9.60	1.2	1.21	0.288
Method*M	8	5.51	0.689	0.696	0.696
θ * M	1	4.72	4.72	4.763	0.029*
Method * θ * M	8	4.48	0.56	0.565	0.807
Residuals	1195	1356.9	1.136		
